# Comparison of oral metabolome profiles of stimulated saliva, unstimulated saliva, and mouth-rinsed water

**DOI:** 10.1038/s41598-021-04612-x

**Published:** 2022-01-13

**Authors:** Yuki Maruyama, Yuichiro Nishimoto, Kouta Umezawa, Ryosuke Kawamata, Yuko Ichiba, Kota Tsutsumi, Mitsuo Kimura, Shinnosuke Murakami, Yasushi Kakizawa, Takashi Kumagai, Takuji Yamada, Shinji Fukuda

**Affiliations:** 1 Research and Development Headquarters, Lion Corporation, 7-2-1 Hirai, Edogawa-ku, Tokyo, 132-0035 Japan; 2 Metabologenomics, Inc., 246-2 Mizukami, Kakuganji, Tsuruoka, Yamagata 997-0052 Japan; 3 Hiyoshi Oral Health Clinics, 2-1-16 Hiyoshi-cho, Sakata, Yamagata 998-0037 Japan; 4 Institute for Advanced Biosciences, Keio University, 246-2 Mizukami, Kakuganji, Tsuruoka, Yamagata 997-0052 Japan; 5 Department of Life Science and Technology, Tokyo Institute of Technology, 2-12-1 Ookayama, Meguro, Tokyo, 152-8550 Japan; 6 Gut Environmental Design Group, Kanagawa Institute of Industrial Science and Technology, 3-25-13 Tonomachi, Kawasaki-ku, Kawasaki, Kanagawa 210-0821 Japan; 7 Transborder Medical Research Center, University of Tsukuba, 1-1-1 Tennodai, Tsukuba, Ibaraki 305-8575 Japan

**Keywords:** Metabolomics, Mass spectrometry, Saliva

## Abstract

Saliva includes a substantial amount of biological information, which has enabled us to understand the relationship between oral metabolites and various oral and systemic disorders. However, collecting saliva using a controlled protocol is time-consuming, making saliva an unsuitable analyte in large cohort studies. Mouth-rinsed water (MW), the water used to rinse the mouth, can be collected easily in less time with less difference between subjects than saliva and could be used as an alternative in oral metabolome analyses. In this study, we investigated the potential of MW collection as an efficient alternative to saliva sample collection for oral metabolome profiling. MW, stimulated saliva, and unstimulated saliva were collected from 10 systemically healthy participants. The samples were subjected to metabolome analysis using capillary electrophoresis time-of-flight mass spectrometry, and the types and amounts of metabolites in the samples were compared. Qualitatively, MW contained the same metabolites as unstimulated and stimulated saliva. While the quantity of the metabolites did not drastically change between the sampling methods, all three reflected individual differences, and the features of MW were the same as those of the unstimulated saliva. Overall, these results suggest that MW may be an appropriate alternative to saliva in oral metabolome profile analysis.

## Introduction

Saliva is secreted by the salivary glands, is always present in the oral cavity, and is known to play an important role in maintaining the oral environment^1–4^. Although saliva consists of more than 99% water, it still contains a wide variety of components (both inorganic and organic and ranging from low to high molecular weight) and plays crucial roles in oral health^3–9^. Given the development of analytical techniques, oral metabolites have been reported in recent years to be associated with various oral diseases, such as periodontal disease and dental caries, as well as systemic diseases, such as cancer, inflammatory bowel diseases, and lifestyle-related diseases^9–11^. Since saliva is believed to be reflective of the oral environment, the usefulness of profiling the oral metabolites in saliva has been suggested previously^12^. However, since saliva collection typically takes approximately 5 min per person, it is critical to reduce individual sampling time when a large number of subjects in studies such as cohort studies, are to be assessed. Therefore, an easier alternative method is necessary to reduce the sample collection time per person. One alternative to saliva is mouth-rinsed water (MW), which takes approximately 10 s per person to collect and has been used as an alternative to saliva in oral microbiome analyses^13,14^. Moreover, the MW method can reduce the burden on the study participants, for instance, by lowering the restraint time, and the sample collection procedure itself is simpler than collecting saliva. On the contrary, a possible limitation of using MW is the inability to assess the actual saliva flow rate for the subjects. It also has the drawback of further diluting different analytes, as several analytes are already more diluted in saliva compared to their concentrations in other body fluids. In addition, MW may not be suitable for some protocols^15^. Even though it is expected that MW could be used for oral metabolite evaluation and profiling, the similarities and differences between MW and saliva have not yet been investigated.

The current study aimed to evaluate the usefulness of MW analysis as an oral metabolite profiling method and to clarify whether MW collection is an efficient alternative to saliva collection. Unstimulated saliva (US), stimulated saliva (SS), and MW were collected from 10 systemically healthy volunteers and the metabolome profiles of the samples collected were examined using capillary electrophoresis time-of-flight mass spectrometry (CE-TOFMS)-based metabolome analysis.

## Results

### Qualitative comparison of the oral metabolome profiles of each sampling method

**Figure 1:**
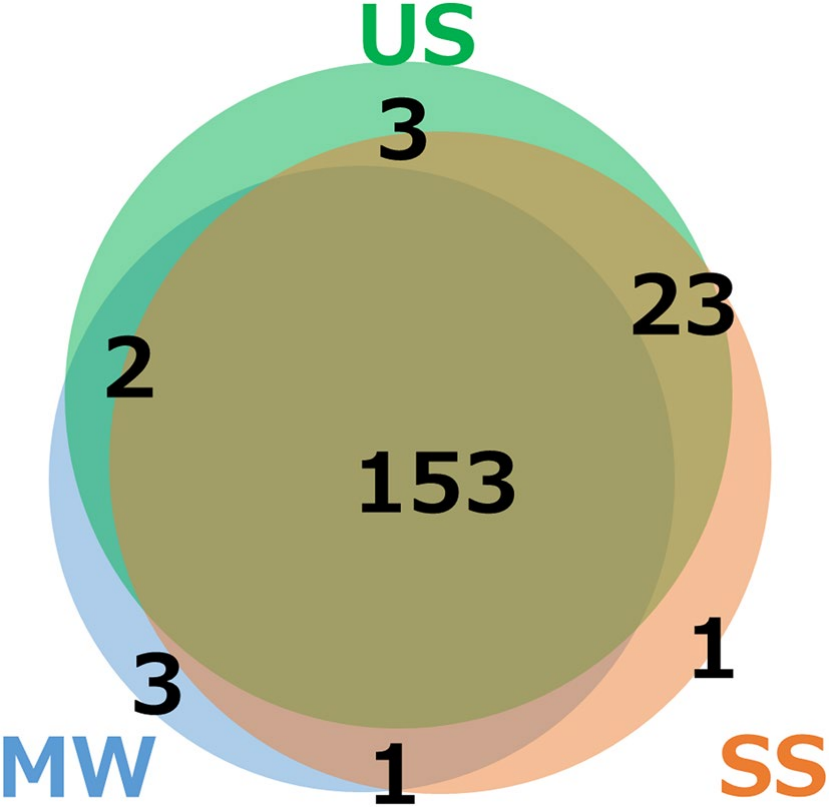
Venn diagram of the number of metabolites detected in each sample collection method. The green circle indicates the number of metabolites detected in the unstimulated saliva (US) samples, the blue circle indicates the number of metabolites detected in the mouth-rinsed water (MW) samples, and the orange circle indicates the number of metabolites detected in the stimulated saliva (SS) samples.

Ten healthy subjects without systemic diseases but with different oral conditions were recruited (Table 1). US, SS, and MW were collected from each subject. The MW samples were collected by allowing subjects to swish their mouth vigorously for 10 s with 3 mL sterilized water and then collecting the rinsed-out water into a specimen tube. Of the 507 metabolites assessed by CE-TOFMS, 212 metabolites were detected in at least one sample from the 10 subjects. After excluding the metabolites detected in only a single sample from a subject, 186 metabolites remained. To qualitatively compare the metabolome profiles of each sampling method, these 186 metabolites were further analyzed. The number of unique metabolites detected in each sampling method and those not detected in each sampling method were counted and are displayed in a Venn diagram (Fig. 1). A total of 153 metabolites (82.3%) were detected in all three sampling methods (US, SS, and MW). Furthermore, the MW samples contained almost the same metabolites as the US and SS samples. Meanwhile, some metabolites were detected in only one or two sampling methods and were not detected in the other methods (Fig. 1 and Table 2). To evaluate the metabolites not detected in the MW samples, we checked the relative area values of these metabolites and observed that these metabolites had small relative area values (Supplementary Fig. S1, Supplementary Tables S3 and S4).

**Table 1:** Study subject details.

Parameters	Study subject									
	B01	B02	B03	B04	B05	B06	B07	B08	B09	B10
Date	Feb 17	Feb 17	Feb 17	Feb 17	Feb 17	Feb 22	Feb 22	Feb 17	Feb 22	Feb 22
Time	15:30	17:20	16:30	17:00	17:00	14:30	14:30	16:00	16:00	16:00
Age	40	39	29	28	30	24	27	32	33	29
Sex	M	F	F	F	F	F	F	M	F	M
DMFT^a^	5	0	4	0	0	0	1	20	9	1
Collection time of Unstimulated Saliva (min)	2	4	2	4	3	6	3	3	5	4
Flow rate of Unstimulated Saliva (g/min)	0.86	0.6	1.04	0.54	0.89	0.22	0.62	1.32	0.37	0.69
Collection time of Stimulated Saliva (min)	1	1	1	1	1	1	1	1	1	1
Flow rate of Stimulated Saliva (g/min)	4.33	3.09	2.75	2.37	4.81	1.45	2.75	3.71	2.73	3.45
PPD^b^ ≧ 4 mm (%)	0	1.8	0	2.7	0	0	0	4.3	7.4	0.8
BOP^c^ (%)	2.7	15.2	6.3	50.9	2.7	3.6	0	0.9	24.1	2.5

^a^Decayed, Missing, and Filled Teeth.

^b^Probing Pocket Depth.

^c^Bleeding On Probing.

**Table 2:** The number of metabolites detected/not detected under each sampling condition.

Sampling method	Number of metabolites detected^a^	Number of metabolites detected only in this method	Number of metabolites not detected only in this method
US^b^	181	3	5
MW^c^	159	3	27
SS^d^	178	1	8
Total^e^	186		

^a^A total of 153 common metabolites were detected in all the sampling methods.

^b^Unstimulated Saliva.

^c^Mouth-rinsed Water.

^d^Stimulated Saliva.

^e^The number of metabolites detected in all samples excluding those detected in only a single study subject.

### Quantitative comparison of the oral metabolome profiles of each sampling method

**Figure 2:**
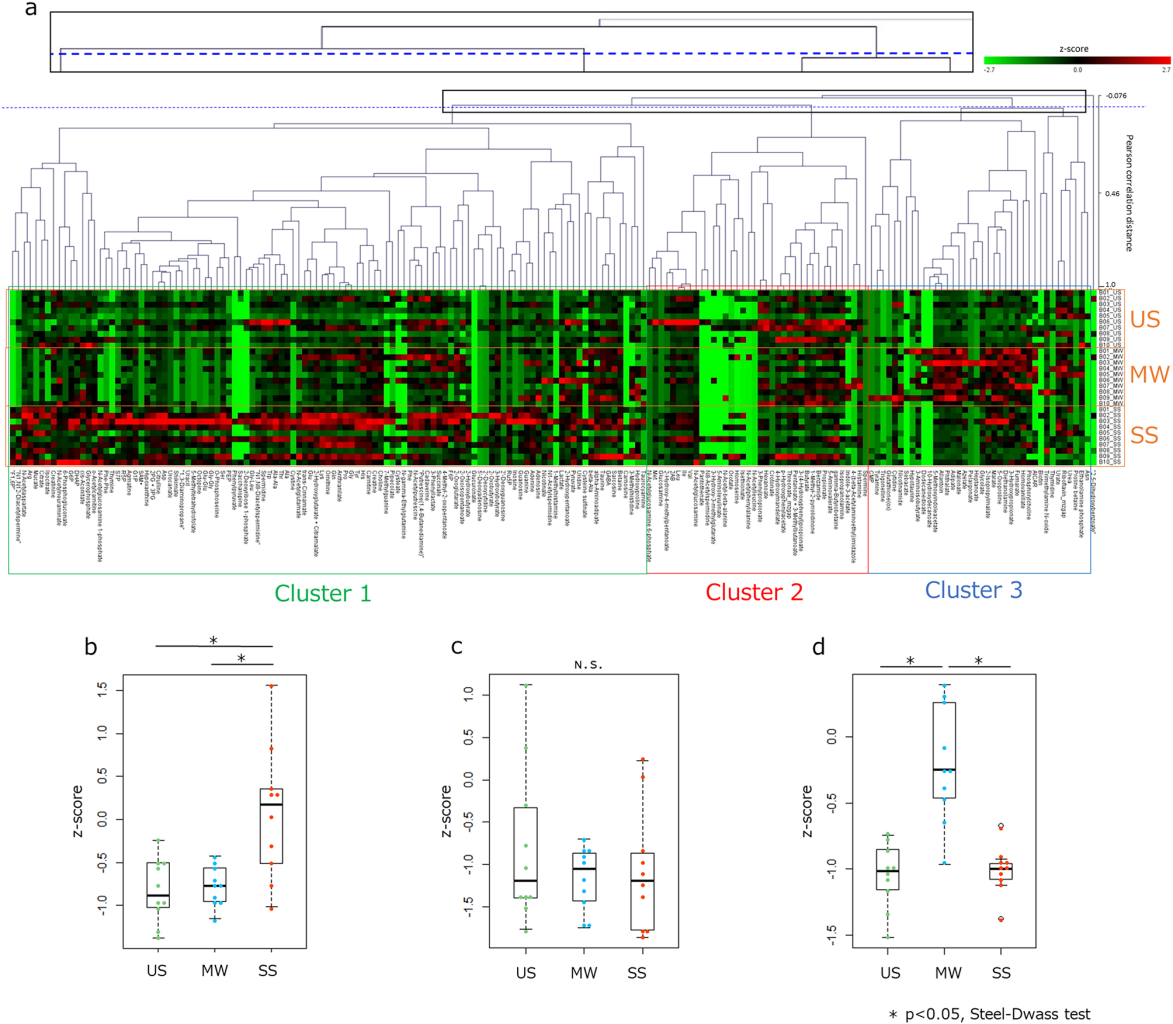
Hierarchical clustering analysis of the oral metabolites obtained using the three sampling methods. The results of quantitative analysis represented by a hierarchical clustering heat map and box plots of z-scores. **(a)** Hierarchical clustering heat map (Pearson correlation distance, average-linkage method). Green box: cluster characteristic of stimulated saliva (SS) samples (Cluster 1); red box: cluster characteristic of all three sampling methods with similar metabolite profiles (Cluster 2); blue box: cluster characteristic of mouth-rinsed water (MW) samples (Cluster 3). (**b–d**) Box plots of average z-scores of all metabolites in each cluster. Z-score was obtained by normalization among the all samples. Each dot represents the z-score corresponding to each subject. (**b)** Cluster 1, (**c)** Cluster 2, (**d)** Cluster 3 (*p < 0.05, Steel–Dwass test).

Hierarchical clustering of the metabolites based on the Pearson correlation distance analysis was performed to quantitatively compare the metabolome profiles of each sampling method (Fig. 2). The results were divided into three major clusters, a cluster characteristic of saliva during stimulation (Cluster 1), a cluster with similarities detected for all sampling methods (Cluster 2), and a cluster characteristic of MW (Cluster 3). Only one metabolite (2, 5-dihydroxybenzoate) was clustered as a separate cluster. To clarify the characteristics of each cluster, we compared the mean z-scores of the metabolites between the different sampling methods within each cluster and found that SS was significantly different in Cluster 1 and MW was significantly different in Cluster 3 when compared with the other sampling methods. Comparing the differences in the sampling methods within each cluster for each metabolite abundance revealed that approximately half or more of the metabolites were not significantly different in any of the clusters; however, some were more abundant in one sampling method or another (Table 3). Among the 38 metabolites in Cluster 3, 15 metabolites were significantly different from those in US or SS. To clarify the characteristics of these 15 metabolites, we analyzed them using public metabolome databases and performed literature searches^16–18^. We found that, in addition to 12 metabolites that have been reported in the saliva, dental calculus, gingival crevicular fluid (GCF), and tongue coating, including dicarboxylic acids and phospholipid metabolites, three metabolites (3-hydroxypropionate, diethanolamine, 10-hydroxydecanoate) were detected characteristically in oral specimens for the first time (Table 4).

**Table 3:** The number of metabolites significantly different in each cluster.

Differences between sampling methods	Number of metabolites		
	Cluster 1	Cluster 2	Cluster 3
Significant difference* US^a^ vs. MW^b^	14	1	13
Significant difference* US vs. SS^c^	38	2	1
Significant difference* SS vs. MW	39	1	12
No significant difference in any comparison	51	34	23

*p < 0.05, Steel–Dwass test.

^a^Unstimulated Saliva.

^b^Mouth-rinsed Water.

^c^Stimulated Saliva.

**Table 4:** Metabolites characteristically detected in mouth-rinsed water.

Metabolite	Category	HMDB^a^	HMDB (saliva)	Dental calculus (GC–MS)^15^	GCF^b^ (GC–MS)^16^	Tongue coating (NMR)^17^
Ethanolamine phosphate	Glycerophospholipid metabolite	〇	〇	〇		
Phosphorylcholine	Glycerophospholipid metabolites (tongue moss metabolites from tongue coating^17^)	〇	〇	〇		〇
Fumarate	Dicarboxylic acids, microbial metabolites (*Aspergillus*)	〇	〇	〇		
3-Hydroxypropionate	Carboxylic acids, microbial metabolites (*Escherichia*, *Klebsiella*, *Saccharomyces*)	〇				
Diethanolamine	Glycerophospholipid metabolite	〇				
5-Oxoproline	Cyclic amino acids (spontaneous cyclization condensation of glutamic acid)	〇	〇	〇		
Malate	Dicarboxylic acid (GCF metabolite^16^)	〇	〇	〇	〇	
Pelargonate	C9 fatty acid	〇	〇	〇		
Malonate	Dicarboxylic acid	〇	〇	〇		
Adipate	Dicarboxylic acid	〇	〇	〇		
Phthalate	Aromatic dicarboxylic acid	〇	〇			
Allantoin	Microbial Metabolites (*Bacillus*, *Streptomyces*)	〇	〇	〇		
5-Methoxyindoleacetate	Indole acetic acid derivative (No report of detection in blood)	〇	〇			
10-Hydroxydecanoate	C10 fatty acids (without HMDB registration)					
Dodecanedioate	Dicarboxylic acid (tartar metabolite^15^)	〇		〇		

^a^Human Metabolome Database.

^b^Gingival Crevicular Fluid.

**Figure 3:**
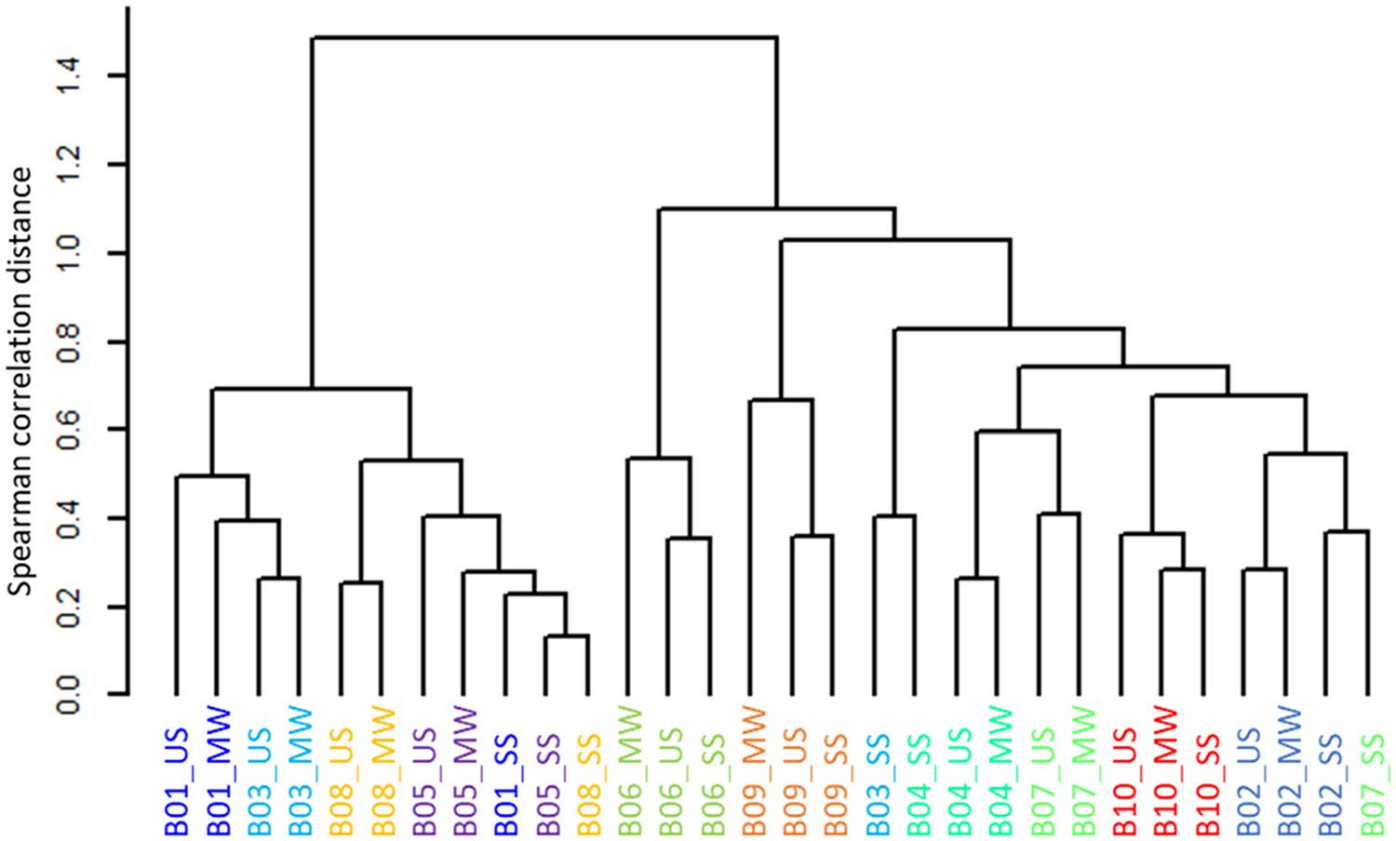
Hierarchical clustering analysis of oral metabolome profiles based on Spearman correlation distance with Ward’s method. A total of 108 metabolites commonly detected in the three sampling methods with no significant difference were used. Samples from the same subject are shown in the same color. US, unstimulated saliva; SS, stimulated saliva; MW, mouth-rinsed water.

**Figure 4:**
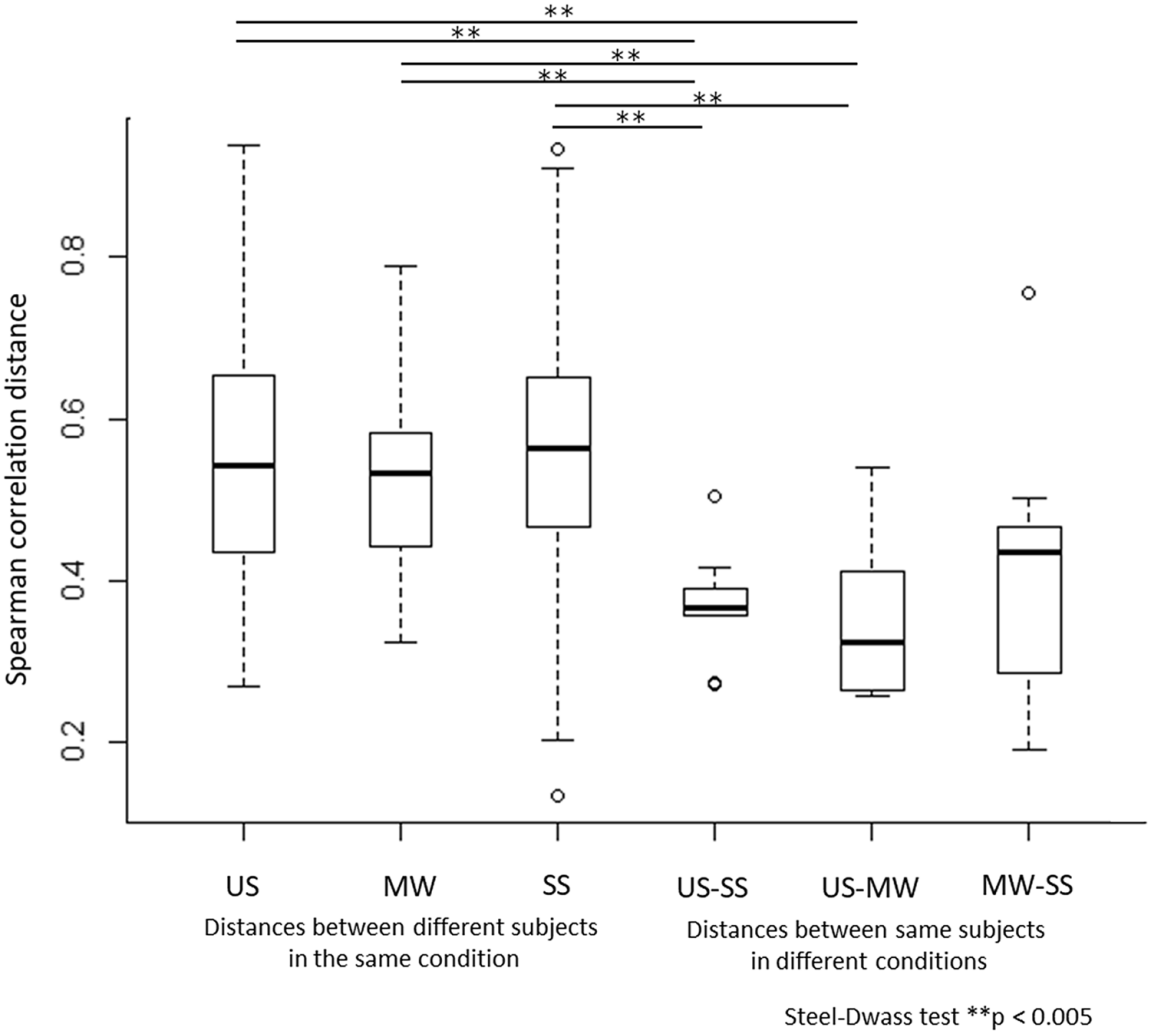
Box plots of Spearman correlation distance between each sampling method and subjects. A total of 108 metabolites commonly detected in the three sampling methods with no significant difference were used. US, unstimulated saliva; SS, stimulated saliva; MW, mouth-rinsed water.

Finally, to clarify whether the oral metabolite composition obtained from the three sampling methods reflected individual differences, 108 metabolites (58%) out of 186 metabolites commonly detected with no significant difference among the three sampling methods were subjected to hierarchical clustering using Spearman correlation distance with Ward's method (Supplementary Table S1). US and MW clustered next to each other in five of the ten study subjects (Fig. 3). We also examined the distance among subjects within the same sampling method and the distance between sampling methods for the same subjects (Fig. 4). This revealed significant differences in the distance among subjects in the same sampling method as well as in the distance between sampling methods for the same subjects in US vs. MW and US vs. SS comparisons.

## Discussion

The results showed that MW qualitatively contained almost the same metabolome information as that of US and SS. Quantitatively, a limited number of metabolites were characteristic of MW and SS, but individual differences were reflected in all three sampling methods (Table 3). These findings suggest that MW is a promising alternative to saliva samples for analyzing oral metabolome profiles.

With respect to qualitative analysis, the composition of metabolites in the MW samples was more than 80% identical to that of the US and SS samples, which have been previously used for oral metabolome analysis. Furthermore, the samples collected using the different methods contained almost the same composition (Fig. 1). Since the diversity of the oral microbiota in US, SS, and MW is similar according to the oral microbiome studies conducted by Jo et al.^13,14^, it can be expected that MW would reflect US and SS metabolome profiles as well. Figueira et al.^19^ reported findings from a comparative analysis of unstimulated, stimulated, and parotid saliva using nuclear magnetic resonance spectroscopy-based metabolome analysis, and detected 45, 44, and 44 metabolites, respectively. Although there are differences in the composition ratios, the metabolites detected are almost identical. This is consistent with our current results and validates the study because the types of metabolites detected in US and SS signified almost the same metabolome information.

The relative area of the metabolites not detected in MW samples was small (Supplementary Fig. S1), suggesting that the MW sampling method failed to detect few metabolites, perhaps due to the dilution of metabolites present at low-concentrations in the oral cavity, resulting in these metabolites not being detected in MW. Metabolites not detected in MW but detected in more than half of the US and SS samples included thymine, octopine, and O-phosphoserine, which are pyrimidine metabolites, a derivative of arginine and alanine, and a phosphate ester of serine, respectively. These metabolites have either not been quantified or detected in saliva or their quantitative values have been reported to not be large^20^. Overall, these results indicate that MW contains almost the same metabolome information as US and SS, but there are some components that are characteristic of only US and SS. These points should be considered when conducting research using MW sampling for oral metabolome analysis.

With respect to quantitative analysis, we observed that the oral metabolites obtained using the three collection methods were divided into three major clusters by hierarchical clustering analysis: clusters characteristic of MW, of SS, and of all three sampling methods with similar metabolome profiles (Fig. 2A, B and Table 3). Cluster 1, a cluster characteristic of SS, contained most of the amino acids (Supplementary Table S2). Figueira et al.^20^ reported that the metabolite compositions of US and SS differ and speculated the reason for the difference to be the increase in the proportion of parotid saliva with masticatory stimulation, which was reflected in the SS composition. In addition, as reported by Neyraud et al.^21^, amino acids are found to be more abundant in SS than in US. In MW, 15 metabolites were characteristic and most of them were considered to have oral origin, i.e., saliva, tartar, GCF, or the tongue coating (Table 4). The reason for the greater abundance of these metabolites in MW compared to that in US or SS is unclear. However, it is possible that these metabolites were more easily obtained by rinsing the mouth with water than by salivation, with or without stimulation. Accordingly, care should be taken when performing comparative analysis of these metabolites, as they may behave differently when compared to those in US or SS.

Hierarchical clustering and comparative analysis of the 108 oral metabolites commonly detected in the three sampling methods with no significant differences (Figs. 3 and 4) revealed that the differences among the subjects were larger than the differences among the three sampling methods. In other words, the profiles of these metabolites reflected individual differences rather than differences depending on the sampling method, suggesting that MW, like US and SS, may be an oral specimen that reflects individual differences. We have reported that the differences in the microbiome reflect individual differences rather than sampling method differences (MW, SS, US, and tongue) in our previous comparative study^13^. We, therefore, believe that similar results were obtained in the current study on metabolites.

The findings of this study must be seen in the light of some limitations. Salivary composition is well known to exhibit circadian rhythms. Kawanishi et al. reported significant differences in metabolite concentrations in unstimulated saliva between morning (8:00–9:00) and evening (17:00–18:00), and in stimulated saliva between morning and daytime (12:00–13:00) and between daytime and evening^22^. In the current study, as the samples were collected between 14:30 and 17:20, it is presumed that the observed differences between the subjects are unlikely to be greatly affected by the diurnal variation. However, its effects cannot be completely ruled out and need to be addressed in future studies. In addition, we profiled hydrophilic metabolites using CE-TOFMS. Since 99% of saliva is water, CE-TOFMS is suitable for profiling a wide range of metabolites. However, poorly water-soluble chemical compounds, such as lipids, have been found to be present in saliva^23,24^. Therefore, further analysis is needed to perform a fully comprehensive oral metabolome profiling. Moreover, although MW appears to be a useful method to collect oral samples from subjects who have difficulty producing saliva, such as patients with dry mouth and the elderly, to verify the usefulness of MW sampling for these individuals, it will be necessary to include subjects with low saliva volume, such as those with xerostomia.

## Conclusions

Our qualitative analysis showed that the MW samples contained the same metabolome information as that of the US and the SS samples. However, our quantitative analysis revealed that MW contained some characteristic metabolites. For the metabolome profiles without obvious sampling methods-based quantitative changes, samples from all three sampling methods reflected individual differences, especially with greater similarities between MW and US. MW samples have previously been reported to be promising alternatives to saliva samples in oral microbiome analysis, and the MW method is also expected to be an effective tool for oral metabolome analysis.

## Methods

### Ethics statement

This study was approved by the ethics committee of Chiyoda Para Medical Care Clinic (Tokyo, Japan, Issuing number: UMIN000031334). All participants understood the purpose of the study and provided informed consent. All experiments were performed in accordance with the approved guidelines.

### Sample collection and dental examination

Sample collection and dental examinations were performed as described previously^13^. Three male and seven female systemically healthy volunteers aged 24 to 40 years. (mean ± s.d., 31.1 ± 4.8 years.) were recruited at the Hiyoshi Oral Health Clinics (Yamagata, Japan). All participants met the inclusion and the exclusion criteria. The inclusion criteria were (a) 20–65 years old and (b) subjects who do not use medicines regularly. The exclusion criteria were: (a) smoker, (b) denture wearer, (c) brace wearer, (d) systemic disease, (e) received antibiotics in the last 6 months, and (f) pregnancy or breastfeeding. Prior to sample collection, the subjects were instructed to not brush their teeth from the previous night to the time of sampling and were prohibited from eating or drinking for at least 1 h prior to sampling. They also received directions on how to rinse their mouth and spit the water, while being careful to keep still when sample was being collected. They did not wash their mouth before the US was collected. For US collection, the saliva accumulated in the mouth was spit out into a Falcon tube every minute until 2 mL or more of it was collected in the tube. The time required for collection was 2–6 min for each subject. After collecting US using the spitting method, the subjects were asked to swish their mouth vigorously for 10 s with 3 mL sterilized water and then spit the MW into a specimen tube once. Following the collection of the MW samples, paraffin-stimulated SS were collected. SS was collected by spitting the saliva that had accumulated in the mouth while chewing Parafilm, into a Falcon tube until 2 mL or more of it was collected in the tube. Each of the 10 subjects took approximately 1 min. The samples were collected during the afternoon consultation hours (14:30–17:30 h). Sampling date and time are as described in Table 1. All samples were placed in a freezer within 10 min of collection and stored at −80 °C until use.

After sample collection, dental examinations were performed and the number of the present, decayed, missing, and filled teeth were recorded. The periodontal pocket depth and bleeding on probing at four sites (mesiobuccal, distobuccal, mesiolingual, and distolingual) of all teeth were measured using a periodontal pocket probe. A summary of the oral health conditions of the subjects was shown in Table 1.

### Metabolome analysis

Metabolome analysis was performed as previously described^25^. Frozen collected samples were thawed and centrifuged at 13,000×*g* for 5 min at 4 °C. The supernatant was transferred to a 5-kDa-cutoff filter (Human Metabolome Technologies, Tsuruoka, Japan) to remove proteins of sizes greater than 5 kDa. Prior to CE-TOFMS analysis, a 45 µL aliquot of the filtrate was added to 5 µL of Milli-Q water containing reference compounds (200 mmol/L each of methionine sulfone, D-camphor-10-sulfonic acid, 3-aminopyrrolidine, and trimesic acid). CE-TOFMS-based metabolome profiling was performed using an Agilent 7100 Capillary Electrophoresis system (Agilent technologies, Waldbronn, Germany), an Agilent 6224 TOF LC/MS system (Agilent technologies, Santa Clara, CA), an Agilent 1200 series isocratic HPLC pump, a G1603A Agilent CE-MS adapter kit, and a G1607A Agilent CE-electrospray ionization (ESI)-MS sprayer kit. In anionic metabolites analysis, ESI sprayer was replaced with a platinum needle instead of an initial stainless-steel needle^25^. Other conditions of the CE–ESI–MS sprayer were the same as received. The metabolome analysis conditions were the same as those described elsewhere^9,26–28^. Data analysis were performed using the metabolome analysis software MasterHands as previously described^29^.

### Data analysis

All quantitative metabolite data were converted to ratios for each metabolite concentration (Supplementary Tables S3 and S4). The Steel–Dwass test was used to compare the ratio of each metabolite concentration in the US, SS, and the MW samples. A *P* value of less than 0.05 was considered statistically significant. The hierarchical clustering heat map analyses were performed using MeV TM4 (ver. 4.9.4; http://mev.tm4.org). All other analyses was performed in R (ver. 3.4.4; R Foundation for Statistical Computing, Vienna, Austria).

## Supplementary Information

Supplementary Information 1.

Supplementary Information 2.

Supplementary Information 3.

## Data Availability

The datasets generated during and/or analyzed during the current study are available from the corresponding author on reasonable request.
