# Supplementary material for: Comparison of oral metabolome profiles of stimulated saliva, unstimulated saliva, and mouth-rinsed water
Source: Sci Rep. 2022 Jan 13;12:689. doi: 10.1038/s41598-021-04612-x (PMC8758762; doi:10.1038/s41598-021-04612-x)
Supplement: Supplementary file 1 — Supplementary Information 1. [file 41598_2021_4612_MOESM1_ESM.pdf]

Comparison of oral metabolome profiles of stimulated saliva, unstimulated saliva, and mouth-rinsed water

Yuki Maruyama[1], Yuichiro Nishimoto[2], Kouta Umezawa[3], Ryosuke Kawamata[1], Yuko Ichiba[1], Kota Tsutsumi[1], Mitsuo Kimura[1], Shinnosuke Murakami[2,4], Yasushi Kakizawa[1], Takashi Kumagai[3], Takuji Yamada[2,5] & Shinji Fukuda[2,4,6,7*]

[1] Research and Development Headquarters, Lion Corporation, 7-2-1 Hirai, Edogawa-ku, Tokyo, 132-0035, Japan.
[2] Metabologenomics, Inc., 246-2 Mizukami, Kakuganji, Tsuruoka, Yamagata, 997-0052, Japan.
[3] Hiyoshi Oral Health Clinics, 2-1-16 Hiyoshi-cho, Sakata, Yamagata, 998-0037, Japan.
[4] Institute for Advanced Biosciences, Keio University, 246-2 Mizukami, Kakuganji, Tsuruoka, Yamagata, 997-0052, Japan.
[5] Department of Life Science and Technology, Tokyo Institute of Technology, 2-12-1 Ookayama, Meguro, Tokyo, 152-8550, Japan.
[6] Intestinal Microbiota Project, Kanagawa Institute of Industrial Science and Technology, 3-25-13 Tonomachi, Kawasaki-ku, Kawasaki, Kanagawa, 210-0821, Japan.
[7] Transborder Medical Research Center, University of Tsukuba, 1-1-1 Tennodai, Tsukuba, Ibaraki, 305-8575, Japan.

*Correspondence:
Shinji Fukuda
Metabologenomics, Inc., 246-2 Mizukami, Kakuganji, Tsuruoka, Yamagata, 997-0052, Japan
Email: sfukuda@metagen.co.jp
Tel: (81+)235-64-0330

Supplementary Table S1. The 108 oral metabolites commonly detected between the three sampling methods with no significant differences in each cluster.

KEGG ID	Compound Name	average±SE			p-value*		
		US[a]	MW[b]	SS[c]	US vs MW	US vs SS	MW vs SS
C11118	1-Methyl-2-pyrrolidinone	1.3±0.26	1.1±0.21	0.91±0.20	0.99	0.36	0.40
C05127	1-Methylhistamine	0.0017±0.000071	0.0033±0.0	0.0037±0.0002	0.88	0.97	0.82
C02356	2-Aminobutyriate	0.040±0.018	0.039±0.0063	0.03±0.0065	0.32	0.95	0.59
C00881	2'-Deoxycytidine	0.0024±0.00046	0.0±0.0	0.0067±0.00086	0.31	0.71	0.16
C00330	2'-Deoxyguanosine	0.0055±0.000067	0.0±0.0	0.018±0.0032	0.31	0.39	0.078
C03264	2-Hydroxy-4-methylpentanoate	0.16±0.056	0.10±0.014	0.097±0.015	0.97	0.87	0.56
–	2-Hydroxypentanoate	0.071±0.019	0.097±0.016	0.071±0.011	0.25	0.73	0.36
C02504	2-Isopropylmalate	0.011±0.0023	0.027±0.0036	0.021±0.0040	0.050	0.086	0.68
C00109	2-Oxobutyrate	0.053±0.014	0.067±0.01	0.092±0.034	0.52	0.32	0.15
C00141	2-Oxoisopentanoate	0.029±0.0049	0.041±0.0023	0.039±0.0042	0.73	0.086	0.68
C01744	3-(4-Hydroxyphenyl)propionate	0.35±0.078	0.43±0.048	0.32±0.057	0.98	0.58	0.53
C05145	3-Aminoisobutyrate	0.018±0.0025	0.030±0.0055	0.019±0.0016	0.77	0.89	0.77
C03761	3-Hydroxy-3-methylglutarate	0.0078±0.00055	0.010±0.0	0.0098±0.00089	0.40	0.74	0.82
C01089	3-Hydroxybutyrate	0.13±0.017	0.24±0.033	0.30±0.11	0.12	0.29	1.0
C01152	3-Methylhistidine	0.0055±0.00046	0.0088±0.00057	0.0073±0.00095	0.63	0.73	0.92
C05607	3-Phenyllactate	0.056±0.010	0.086±0.011	0.095±0.016	0.14	0.77	0.92
C05629	3-Phenylpropionate	0.56±0.11	0.51±0.090	0.37±0.067	0.97	0.40	0.36
C05135	4-(beta-Acetylaminoethyl)imidazole	0.0032±0.00047	0.0069±0.000089	0.0035±0.00038	0.98	0.92	0.97
C11527	4-Hydroxymandelate	0.22±0.065	0.19±0.04	0.18±0.061	0.97	0.82	0.84
C00233	4-Methyl-2-oxopentanoate	0.050±0.010	0.071±0.0075	0.084±0.012	0.12	0.05	0.77
C00430	5-Aminolevulinate	0.0032±0.00022	0.0±0.0	0.0042±0.0	0.31	0.88	0.58
C00431	5-Aminovalerate	9.4±1.6	8.8±1.4	7.5±1.4	1.0	0.82	0.82
C05198	5'-Deoxyadenosine	0.0024±0.000029	0.0±0.0	0.0041±0.00075	0.31	0.82	0.16
C02242	7-Methylguanine	0.0039±0.00025	0.0060±0.00034	0.0064±0.00059	0.46	0.80	0.33
C00212	Adenosine	0.0048±0.00093	0.0091±0.0015	0.0072±0.0012	0.36	0.22	0.95
C04677	AICAR[fd]	0.032±0.0015	0.066±0.0030	0.036±0.002	0.83	0.96	0.57
C00041	Alanine	0.62±0.23	0.62±0.079	1.1±0.23	0.49	0.25	0.49
C00993	Alanyl-alanine	0.021±0.0059	0.029±0.0033	0.044±0.0090	0.22	0.086	0.54
C00152	Asparagine	0.031±0.00074	0.0±0.0	0.00074±0.0	0.31	0.75	0.58
C08261	Azelate	0.0063±0.0010	0.024±0.0024	0.0093±0.0017	0.19	0.97	0.20
C09815	Benzamide	0.39±0.066	0.49±0.055	0.34±0.041	0.77	0.77	0.27
C00099	beta-Alanine	0.037±0.0034	0.050±0.004	0.039±0.0067	0.086	0.86	0.19
C00719	Betaine	0.096±0.0091	0.14±0.016	0.19±0.037	0.060	0.050	0.54
C00120	Biotin	0.025±0.0030	0.040±0.0	0.027±0.0	0.40	0.30	1.0
C01672	Cadaverine	0.28±0.085	0.30±0.088	0.28±0.078	0.92	0.97	0.92
C00386	Carnosine	0.0048±0.0	0.0±0.0	0.0064±0.00069	0.58	0.43	0.16
C00417	cis-Aconitate	0.014±0.0035	0.025±0.0031	0.024±0.0042	0.19	0.36	0.89
C00158	Citrate	0.43±0.11	0.18±0.041	0.63±0.17	0.16	0.82	0.4
C00055	Cytidine-5'-monophosphate	0.0±0.0	0.026±0.0	0.0093±0.0011	0.58	0.31	0.88
C00791	Creatinine	0.12±0.011	0.13±0.011	0.16±0.012	0.92	0.32	0.36
C00506	Cysteate	0.015±0.0017	0.032±0.00069	0.022±0.0023	1.0	0.31	0.87
C00606	Cysteine sulfinate	0.041±0.0040	0.083±0.0047	0.073±0.0059	0.91	0.97	0.96
C00491	Cystine	0.0064±0.00062	0.0±0.0	0.0094±0.0015	0.078	0.96	0.078
C00475	Cytidine	0.013±0.0024	0.020±0.0034	0.014±0.0017	0.086	0.59	0.40
C00092	Glucose 6-phosphate	0.061±0.019	0.092±0.0099	0.14±0.029	0.29	0.14	0.77
C00334	gamma-Aminobutyrate	0.031±0.0043	0.042±0.0050	0.040±0.0082	0.29	0.92	0.86
C01181	gamma-Butyrobetaine	0.053±0.011	0.049±0.0045	0.060±0.011	0.92	0.82	0.82
C00329	Glucosamine	0.083±0.0	0.0±0.0	0.025±0.0031	0.58	0.40	0.078
C00191	Glucuronate	0.0±0.0	0.0±0.0	0.028±0.0019	N.A.	0.16	0.16

C00127	Glutathione(ox)	0.0032±0.0	0.010±0.0011	0.0026±0.0	0.21	1.0	0.21
C00037	Glycine	2.0±0.52	2.1±0.42	3.7±0.65	0.59	0.12	0.25
C00093	Glycerophosphate	0.25±0.062	0.22±0.019	0.26±0.035	0.89	0.77	0.95
C00160	Glycolate	0.20±0.019	0.32±0.038	0.22±0.020	0.050	0.68	0.072
C17714	Heptanoate	0.0089±0.0015	0.027±0.0039	0.017±0.0	0.18	0.88	0.11
C01585	Hexanoate	0.53±0.13	0.50±0.085	0.31±0.060	0.99	0.59	0.29
C00388	Histamine	0.023±0.0094	0.063±0.018	0.022±0.0069	0.92	0.97	0.77
C00263	Homoserine	0.014±0.0011	0.025±0.0	0.015±0.0015	0.23	1.0	0.23
C05582	Homovanillate	0.13±0.026	0.20±0.029	0.15±0.028	0.22	0.86	0.40
C01015	Hydroxyproline	0.018±0.0028	0.034±0.0041	0.026±0.0042	0.97	0.86	0.99
C00519	Hypotaurine	0.041±0.0046	0.10±0.0075	0.039±0.0019	1.0	0.62	0.71
C00407	Isoleucine	0.14±0.060	0.060±0.014	0.14±0.035	0.77	0.89	0.16
C00954	Indole-3-acetate	0.054±0.0069	0.082±0.0049	0.040±0.00086	0.74	0.33	0.98
C05771	Isopropanolamine	0.017±0.0027	0.017±0.0013	0.011±0.0015	1.0	0.16	0.072
C00123	Leucine	0.36±0.12	0.17±0.040	0.34±0.073	0.45	0.89	0.25
C00073	Methionine	0.28±0.099	0.073±0.0043	0.071±0.014	1.0	0.32	0.20
C00612	N1-Acetylspermidine	0.011±0.0032	0.020±0.0043	0.023±0.0062	1.0	0.54	0.72
C01029	N8-Acetylspermidine	0.0069±0.0	0.0±0.0	0.0059±0.00028	0.58	0.88	0.31
C01073	N-Acetyl-beta-alanine	0.021±0.0012	0.043±0.0	0.031±0.0016	0.12	0.87	0.63
C00357	N-Acetylglucosamine 6-phosphate	0.030±0.0077	0.0±0.0	0.0±0.0	0.31	0.31	N.A.
C00624	N-Acetylglutamate	0.0064±0.00092	0.013±0.0013	0.013±0.0023	0.72	0.050	0.081
C02710	N-Acetylleucine	0.0056±0.00073	0.0±0.0	0.0±0.0	0.16	0.16	N.A.
C00270	N-Acetylneuraminate	1.7±0.31	2.0±0.49	2.3±0.54	1.0	0.68	0.54
C03519	N-Acetylphenylalanine	0.0077±0.00080	0.013±0.0	0.0±0.0	0.63	0.16	0.58
C02714	N-Acetylputrescine	0.040±0.0065	0.046±0.0071	0.068±0.011	0.82	0.22	0.29
C01047	N-gamma-Ethylglutamine	0.011±0.0011	0.017±0.0014	0.013±0.0018	0.92	0.94	0.98
C00153	Nicotinamide	0.013±0.0031	0.024±0.0017	0.013±0.0014	0.77	0.56	0.18
C00253	Nicotinate	0.051±0.0047	0.24±0.087	0.098±0.019	0.68	0.16	0.12
C02571	o-Acetylcarnitine	0.020±0.0026	0.023±0.00082	0.03±0.0046	0.072	0.06	0.64
C00295	Orotate	0.026±0.0030	0.040±0.0	0.049±0.0048	0.23	0.67	0.11
C00864	Pantothenate	0.0070±0.00069	0.0±0.0	0.0089±0.00095	0.31	0.77	0.16
C00803<br>+C08262	Pentanoate<br>+ 3-Methylbutanoate[e]	0.56±0.086	0.54±0.060	0.44±0.088	0.95	0.54	0.32
C00079	Phenylalanine	0.55±0.099	0.57±0.096	0.62±0.11	0.97	0.95	0.95
C00166	Phenylpyruvate	0.028±0.0	0.0±0.0	0.058±0.0047	0.58	0.43	0.16
C00642	p-Hydroxyphenylacetate	0.42±0.10	0.36±0.045	0.37±0.11	0.92	0.81	0.95
C00148	Proline	1.2±0.37	1.2±0.36	2.9±0.56	1.0	0.086	0.12
C10172	Proline betaine	0.010±0.0028	0.017±0.0035	0.013±0.0017	0.95	0.99	0.92
C00163	Propionate	12.0±2.8	11.0±1.6	8.1±1.8	1.0	0.64	0.32
C00134	Putrescine(1,4-Butanediamine)	1.6±0.30	1.4±0.22	1.9±0.31	1.0	0.86	0.40
C00255	Riboflavin	0.18±0.039	0.22±0.033	0.17±0.023	0.95	0.95	0.54
C05382	Sedoheptulose 7-phosphate	0.034±0.0024	0.049±0.0031	0.091±0.015	1.0	0.082	0.082
C00449	Saccharopine	0.007±0.0005	0.0±0.0	0.013±0.00086	0.16	0.59	0.078
C00213	Sarcosine	0.16±0.018	0.2±0.022	0.19±0.039	0.45	1.0	0.86
C08277	Sebacate	0.0059±0.0	0.0058±0.00047	0.0±0.0	0.49	0.58	0.16
C00750	Spermine	0.0076±0.0017	0.0±0.0	0.0±0.0	0.31	0.31	N.A.
C00042	Succinate	1.6±0.37	1.8±0.33	1.7±0.43	0.89	1.0	0.95
C01959	Taurocyamine	0.17±0.034	0.31±0.020	0.20±0.041	0.82	0.83	1.0
C00188	Threonine	0.10±0.048	0.078±0.0088	0.14±0.028	0.68	0.22	0.25
C01620	Threonate	0.38±0.094	0.34±0.057	0.31±0.071	0.99	0.92	0.68
C00214	Thymidine	0.078±0.011	0.20±0.00037	0.11±0.0016	0.98	0.95	0.97
C01004	Trigonelline	0.0098±0.0	0.015±0.0	0.0064±0.0010	1.0	0.88	0.88
C01104	Trimethylamine N-oxide	0.015±0.0058	0.035±0.012	0.018±0.0067	0.62	0.73	0.29
C00078	Tryptophan	0.036±0.0093	0.026±0.0037	0.042±0.0074	0.94	0.84	0.47
C00082	Tyrosine	0.56±0.097	0.59±0.075	1±0.15	1.0	0.12	0.14
C00483	Tyramine	0.027±0.0079	0.046±0.014	0.025±0.0013	0.99	1.0	1.0

C00366	Urate	6.6±1.2	6.9±1.1	6.7±1.1	1.0	0.99	1.0
C00086	Urea	36.0±7.2	28.0±4.7	25.0±8.2	0.64	0.59	0.73
C00299	Uridine	0.15±0.026	0.26±0.034	0.17±0.022	0.66	0.25	0.82
C00183	Valine	0.46±0.19	0.25±0.063	0.35±0.081	1.0	0.95	0.68

*Steel-Dwass test
[a]Unstimulated Saliva
[b]Mouth-rinsed Water
[c]Stimulated Saliva
[d]5-Aminoimidazole-4-carboxamide ribonucleotide
[e]For pentanoate and 3-Methylbutanoate, the peaks were not sufficiently separated in the CE-TOFMS analysis, so the two metabolites were quantified together.

Supplementary Table S2. List of 186 metabolites used for quantitative analysis.

KEGG ID	Compound Name	Classification[a]	Cluster
C04137	Octopine	2-Heteroaryl carboxamides	Cluster1
C00019	S-Adenosylmethionine	5'-Deoxyribonucleosides	Cluster1
C05198	5'-Deoxyadenosine	5'-Deoxyribonucleosides	Cluster1
-	N1,N8-Diacetylspermidine	Acetamides	Cluster1
C02571	o-Acetylcarnitine	Acyl carnitines	Cluster1
C00719	Betaine	Alpha amino acids	Cluster1
C01005	O-Phosphoserine	Alpha amino acids	Cluster1
C00077	Ornithine	Alpha amino acids	Cluster1
C00408	Pipecolate	Alpha amino acids	Cluster1
C00213	Sarcosine	Alpha amino acids	Cluster1
C05984	2-Hydroxybutyrate	Alpha hydroxy acids	Cluster1
C00186	Lactate	Alpha hydroxy acids	Cluster1
C00022	Pyruvate	Alpha keto acids	Cluster1
C00986	1,3-Diaminopropane	Amines	Cluster1
C05127	1-Methylhistamine	Amines	Cluster1
C01672	Cadaverine	Amines	Cluster1
C00134	Putrescine(1,4-Butanediamine)	Amines	Cluster1
C00315	Spermidine	Amines	Cluster1
C01152	3-Methylhistidine	Amino acid derivatives	Cluster1
C00956	alpha-Aminoadipate	Amino acid derivatives	Cluster1
C00327	Citrulline	Amino acid derivatives	Cluster1
C00300	Creatine	Amino acid derivatives	Cluster1
C00791	Creatinine	Amino acid derivatives	Cluster1
C00506	Cysteate	Amino acid derivatives	Cluster1
C00606	Cysteine sulfinate	Amino acid derivatives	Cluster1
C00491	Cystine	Amino acid derivatives	Cluster1
C01015	Hydroxyproline	Amino acid derivatives	Cluster1
C01042	N-Acetylaspartate	Amino acid derivatives	Cluster1
C00624	N-Acetylglutamate	Amino acid derivatives	Cluster1
C01047	N-gamma-Ethylglutamine	Amino acid derivatives	Cluster1
C00449	Saccharopine	Amino acid derivatives	Cluster1
C00041	Alanine	Amino acids	Cluster1
C00062	Arginine	Amino acids	Cluster1
C00049	Aspartate	Amino acids	Cluster1
C00064	Glutamine	Amino acids	Cluster1
C00025	Glutamate	Amino acids	Cluster1
C00037	Glysine	Amino acids	Cluster1
C00135	Histidine	Amino acids	Cluster1
C00047	Lysine	Amino acids	Cluster1
C00079	Phenylalanine	Amino acids	Cluster1
C00148	Proline	Amino acids	Cluster1
C00065	Serine	Amino acids	Cluster1
C00188	Threonine	Amino acids	Cluster1
C00078	Tryptophan	Amino acids	Cluster1
C00082	Tyrosine	Amino acids	Cluster1
C00108	Anthranilate	Aminobenzoic acids	Cluster1
C00099	beta-Alanine	Beta amino acids	Cluster1
C01089	3-Hydroxybutyrate	Beta hydroxy acids	Cluster1
C03413	N1,N12-Diacetylspermine	Carboximidic acids	Cluster1
C00612	N1-Acetylspermidine	Carboximidic acids	Cluster1
C02714	N-Acetylputrescine	Carboximidic acids	Cluster1
C00318	Carnitine	Carnitines	Cluster1
C00114	Choline	Cholines	Cluster1
C00423	trans-Cinnamate	Cinnamic acids	Cluster1

C00042	Succinate	Dicarboxylic acids	Cluster1
C00993	Alanyl-alanine	Dipeptides	Cluster1
C01425	Glutamyl-glutamate	Dipeptides	Cluster1
C02037	Glycyl-glycine	Dipeptides	Cluster1
C02155	Glycyl-Leucine	Dipeptides	Cluster1
-	Phenylalanyl-phenylalanine	Dipeptides	Cluster1
C00334	Gamma-Aminobutyric acid	Gamma amino acids	Cluster1
C00191	Glucuronate	Glucuronic acid derivatives	Cluster1
C00879	Mucate	Glucuronic acid derivatives	Cluster1
C00093	Glycerophosphate	Glycerophosphates	Cluster1
C00179	Agmatine	Guanidines	Cluster1
C00354	Fructose 1,6-bisphosphate	Hexose phosphates	Cluster1
C00085	Fructose 6-phosphate	Hexose phosphates	Cluster1
C00092	Glucose 6-phosphate	Hexose phosphates	Cluster1
C05382	Sedoheptulose 7-phosphate	Hexose phosphates	Cluster1
C00386	Carnosine	Hybrid peptides	Cluster1
-	2-Hydroxypentanoate	Hydroxy fatty acids	Cluster1
C00178	Thymine	Hydroxypyrimidines	Cluster1
C02242	7-Methylguanine	Hypoxanthines	Cluster1
C00262	Hypoxanthine	Hypoxanthines	Cluster1
C00785	Urocanate	Imidazoles	Cluster1
C00345	6-Phosphogluconate	Monosaccharide phosphates	Cluster1
C00111	Dihydroxyacetone phosphate	Monosaccharide phosphates	Cluster1
C00103	Glucose 1-phosphate	Monosaccharide phosphates	Cluster1
C04501	N-Acetylglucosamine 1-phosphate	N-acyl-alpha-hexosamines	Cluster1
C00270	N-Acetylneuraminate	N-acylneuraminic acids	Cluster1
C00245	Taurine	Organosulfonic acids	Cluster1
C01959	Taurocyamine	Organosulfonic acids	Cluster1
C00117	Ribose 5-phosphate	Pentose phosphates	Cluster1
C00199	Ribulose 5-phosphate	Pentose phosphates	Cluster1
C00672	2-Deoxyribose 1-phosphate	Pentoses	Cluster1
C05607	3-Phenyllactate	Phenylpropanoic acids	Cluster1
C00166	Phenylpyruvate	Phenylpyruvic acid derivatives	Cluster1
C00074	Phosphoenolpyruvate	Phosphate esters	Cluster1
C00212	Adenosine	Purine nucleosides	Cluster1
C00387	Guanosine	Purine nucleosides	Cluster1
C00330	2'-Deoxyguanosine	Purine nucleosides	Cluster1
C00294	Inosine	Purine nucleosides	Cluster1
C00242	Guanine	Purines	Cluster1
C00147	Adenine	Purines	Cluster1
C00253	Nicotinate	Pyridinecarboxylic acids	Cluster1
C00881	2'-Deoxycytidine	Pyrimidine nucleosides	Cluster1
C00299	Uridine	Pyrimidine nucleosides	Cluster1
C00106	Uracil	Pyrimidines	Cluster1
C00493	Shikimate	Shikimic acids and derivatves	Cluster1
C02630 + C00815	2-Hydroxyglutarate + Citramalate*	Short-chain hydroxy acids + Hydroxy fatty acids	Cluster1
C00109	2-Oxobutyrate	Short-chain keto acids	Cluster1
C00026	2-Oxoglutarate	Short-chain keto acids	Cluster1
C00141	2-Oxoisopentanoate	Short-chain keto acids	Cluster1
C00233	4-Methyl-2-oxopentanoate	Short-chain keto acids	Cluster1
C00631 + C00197	2-Phosphoglycerate + 3-Phosphoglycerate*	Sugar acids	Cluster1
C00440	5-Methyltetrahydrofolate	Tetrahydrofolic acids	Cluster1
C00417	cis-Aconitate	Tricarboxylic acids	Cluster1
C00158	Citrate	Tricarboxylic acids	Cluster1

C00311	Isocitrate	Tricarboxylic acids	Cluster1
C00140	N-Acetylglucosamine	Acylaminosugars	Cluster2
C00357	N-Acetylglucosamine 6-phosphate	Acylaminosugars	Cluster2
C02356	2-Aminobutyrate	Alpha amino acids	Cluster2
C00263	Homoserine	Alpha amino acids	Cluster2
C00388	Histamine	Amines	Cluster2
C05771	Isopropanolamine	Amines	Cluster2
C00750	Spermine	Amines	Cluster2
C00430	5-Aminolevulinate	Amino acid derivatives	Cluster2
C00431	5-Aminovalerate	Amino acid derivatives	Cluster2
C02710	N-Acetylleucine	Amino acid derivatives	Cluster2
C03519	N-Acetylphenylalanine	Amino acid derivatives	Cluster2
C00407	Isoleucine	Amino acids	Cluster2
C00123	Leucine	Amino acids	Cluster2
C00073	Methionine	Amino acids	Cluster2
C00183	Valine	Amino acids	Cluster2
C09815	Benzamide	Benzamides	Cluster2
C01029	N8-Acetylspermidine	Carboximidic acids	Cluster2
C01073	N-Acetyl-beta-alanine	Carboxylic acids	Cluster2
C00163	Propionate	Carboxylic acids	Cluster2
C00329	Glucosamine	Hexoses	Cluster2
C03264	2-Hydroxy-4-methylpentanoate	Hydroxy fatty acids	Cluster2
C03761	3-Hydroxy-3-methylglutarate	Hydroxy fatty acids	Cluster2
C00954	Indole-3-acetate	Indole-3-acetic acid derivatives	Cluster2
C05135	4-(beta-Acetylaminoethyl)imidazole	N-acetyl-2-arylethylamines	Cluster2
C05123	Isethionate	Organosulfonic acids	Cluster2
C11527	4-Hydroxymandelate	Phenols	Cluster2
C00642	p-Hydroxyphenylacetate	Phenols	Cluster2
C01744	3-(4-Hydroxyphenyl)propionate	Phenylpropanoic acids	Cluster2
C05629	3-Phenylpropionate	Phenylpropanoic acids	Cluster2
C00295	Orotate	Pyrimidinecarboxylic acids	Cluster2
C11118	1-Methyl-2-pyrrolidinone	Pyrrolidones	Cluster2
C00864	Pantothenate	Secondary alcohols	Cluster2
C00246	Butyrate	Straight chain fatty acids	Cluster2
C01771	Crotonate	Straight chain fatty acids	Cluster2
C01181	gamma-Butyrobetaine	Straight chain fatty acids	Cluster2
C01585	Hexanoate	Straight chain fatty acids	Cluster2
C00803 + C08262	Pentanoate + 3-Methylbutanoate[*]	Straight chain fatty acids + Methyl-branched fatty acids	Cluster2
C01620	Threonate	Sugar acids	Cluster2
C04677	AICAR[b]	1-Ribosyl-imidazolecarboxamides	Cluster3
C01004	Trigonelline	Alkaloids and derivatives	Cluster3
C00160	Glycolate	Alpha hydroxy acids	Cluster3
C06772	Diethanolamine	Amines	Cluster3
C01879	5-Oxoproline	Amino acid derivatives	Cluster3
C10172	Proline betaine	Amino acid derivatives	Cluster3
C00152	Asparagine	Amino acids	Cluster3
C01104	Trimethylamine N-oxide	Aminoxides	Cluster3
C01606	Phthalate	Benzoic acids	Cluster3
C05145	3-Aminoisobutyrate	Beta amino acids	Cluster3
C01013	3-Hydroxypropionate	Beta amino acids	Cluster3
C00711	Malate	Beta hydroxy acids	Cluster3
C00120	Biotin	Biotin and derivatives	Cluster3
C00122	Fumarate	Dicarboxylic acids	Cluster3
C00383	Malonate	Dicarboxylic acids	Cluster3
C00255	Riboflavin	Flavins	Cluster3

C02504	2-Isopropylmalate	Hydroxy fatty acids	Cluster3
C01551	Allantoin	Imidazoles	Cluster3
C05660	5-Methoxyindoleacetate	Indole-3-acetic acid derivatives	Cluster3
C06104	Adipate	Medium-chain fatty acids	Cluster3
C17714	Heptanoate	Medium-chain fatty acids	Cluster3
C01601	Pelargonate	Medium-chain fatty acids	Cluster3
C08277	Sebacate	Medium-chain fatty acids	Cluster3
C08261	Azelate	Medium-chain fatty acids	Cluster3
C02678	Dodecanedioate	Medium-chain fatty acids	Cluster3
C02774	10-Hydroxydecanoate	Medium-chain hydroxy acids	Cluster3
C00153	Nicotinamide	Nicotinamides	Cluster3
C00127	Glutathione(ox)	Peptides	Cluster3
C00483	Tyramine	Phenethylamines	Cluster3
C05582	Homovanillate	Phenols	Cluster3
C00588	Phosphorylcholine	Phosphocholines	Cluster3
C00346	Ethanolamine phosphate	Phosphoethanolamines	Cluster3
C00475	Cytidine	Pyrimidine nucleosides	Cluster3
C00214	Thymidine	Pyrimidine nucleosides	Cluster3
C00055	Cytidine monophosphate	Pyrimidine ribonucleotides	Cluster3
C00519	Hypotaurine	Sulfinic acids	Cluster3
C00086	Urea	Ureas	Cluster3
C00366	Urate	Xanthines	Cluster3
C00628	2,5-Dihydroxybenzoate	Benzoic acids	—

* For these metabolites, the two peaks were not sufficiently separated in the CE-TOFMS analysis, so the two metabolites were quantified together.

[A]Classified by Human Metabolome Database

[b]5-Aminoimidazole-4-carboxamide ribonucleotide

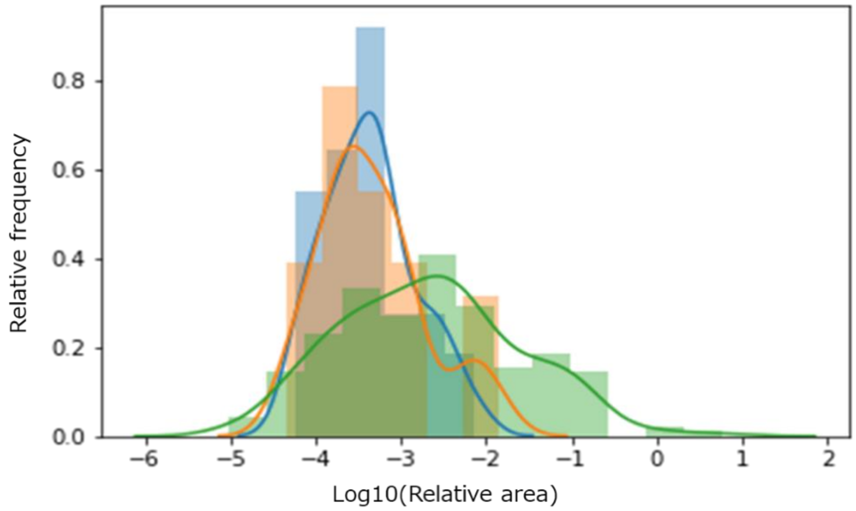

Supplementary Figure S1. Histogram and kernel density estimation of relative area values of oral metabolites. The blue bar represents the metabolites detected in unstimulated saliva (US) samples but not detected in mouth-rinsed water (MW) samples; the orange bar represents the metabolites detected in stimulated saliva (SS) samples but not detected in MW samples; and the green bar represents the frequency of all metabolites detected.
