# Supplementary material for: Comparison of oral metabolome profiles of stimulated saliva, unstimulated saliva, and mouth-rinsed water
Source: Sci Rep. 2022 Jan 13;12:689. doi: 10.1038/s41598-021-04612-x (PMC8758762; doi:10.1038/s41598-021-04612-x)
Supplement: Supplementary file 2 — Supplementary Information 2. [file 41598_2021_4612_MOESM2_ESM.pdf]

Supplementary Table S3. Metabolite concentrations of unstimulated saliva (US), stimulated saliva (SS), and mouth-rinsed water (MW) samples (μM).

KEGG ID	Compound Name	Ave m/z	Ave CorMT	US (uM)										MW (uM)										SS (uM)									
				B01	B02	B03	B04	B05	B06	B07	B08	B09	B10	B01	B02	B03	B04	B05	B06	B07	B08	B09	B10	B01	B02	B03	B04	B05	B06	B07	B08	B09	B10
C00086	Urea	61.0404	33.0	1079	692	601	1140	1359	8	100	1339	694	820	201	81	190	177	453	80.2	130	423	157	257	1071	45.7	34.6	249	1433	65.0	505	1291	68.2	365
C00481	Pyrazole	69.0444	10.9																														
C05670	3-Aminopropionitrile	71.0607	10.0																														
C02294	Methylguanidine	74.0713	9.7																														
C02787	Isobutylamine	74.0969	11.0						2.9										0.63										1.2				
C00986	1,3-Diaminopropane	75.0911	6.9		0.57	0.62	0.45		4.7	0.9		0.48	0.28	0.53	0.29		0.33		0.36				0.6	0.52	1.8	2.9	3.7	0.50	1.4	0.54	0.36	1.8	1.5
C00037	Gly	76.0396	13.0	21.9	168	14.7	36.1	7.9	135	98	15.9	48.3	27.9	16.4	53.0	5.1	20.7	8.4	11.2	13.0	12.7	11.0	22.4	30.4	127	54.5	74.5	14.7	106	111	26.6	133	60.2
C01104	Trimethylamine N-oxide	76.0758	10.3	0.20	0.35	0.072	0.32	0.14		0.089	1.2				0.11			0.13			0.8			0.25	0.30	0.11	0.37	0.20		0.09	1.5		0.075
C05771	Isopropanolamine	76.0759	11.0	0.21	0.51	0.17	0.65	0.13	2.0	0.45	0.25	0.69	0.21	0.12	0.16		0.16	0.13	0.19	0.13	0.13	0.11	0.13	0.14	0.27		0.31		0.34	0.13	0.16	0.49	0.13
C07044	Hydroxyurea	77.0342	34.4																														
C01008	Trimethylsulfonium	77.0422	9.1																														
C01678	Cysteamine	78.0366	10.0																														
C02640	Isoamylamine	88.1124	11.7																														
C00134	Putrescine(1,4-Butanediamine)	89.1076	7.3	16.2	99	15.1	46.2	9.3	163	61.0	16.2	34.9	21.3	8.6	27.8	2.4	15.5	5.3	9.7	9.3	11.0	6.0	19.9	16.8	75.8	28.5	55.5	10.5	51.7	39.0	14.2	48.2	31.1
C00099	beta-Ala	90.0551	11.5	0.82	1.2	0.77	1.0	0.32	2.7	0.85	0.70	1.1	0.56	0.41	0.46	0.19	0.37	0.25	0.44	0.19	0.7	0.38	0.6	0.55	0.56	0.89	1.1	0.25	0.62	0.35	0.82	1.6	0.71
C00041	Ala	90.0551	14.2	7.1	23.9	6.7	15.0	2.9	162	7.7	4.5	14.1	5.6	4.4	8.2	1.9	7.1	2.6	8.4	1.8	4.7	4.9	5.7	8.8	33.4	21.7	37.2	5.7	39.6	6.4	9.7	50.7	15.6
C00213	Sarcosine	90.0551	14.8	2.3	5.5	2.0	6.0	1.2	12.8	4.3	2.0	6.2	2.4	1.1	2.2	0.7	2.9	1.0	1.8	1.0	1.5	1.8	1.6	1.4	4.1	2.1	6.9	1.0	6.6	2.7	1.7	10.8	1.9
C06057	3-Aminopropane-1,2-diol	92.0711	11.5																														
C00292	Aniline	94.0653	11.2																														
C05562	4-Hydroxymethylimidazole	99.0553	10.9																														
C11118	1-Methyl-2-pyrrolidinone	100.0758	33.4	5.7	62.7	9.6	28.7	16.8	135	39.2	15.3	69.6	13.7	2.4	13.6	1.0	6.7	6.6	9.9	7.9	11.6	10.1	30.8	3.2	25.7	6.7	15.1	14.8	26.2	10.6	8.5	63.2	20.0
C00571	Cyclohexylamine	100.1123	12.0																														
C01234	1-Aminocyclopropane-1-carboxylate	102.0548	13.9																														
C00576	Betaine aldehyde	102.0901	11.7																														
C00750	Spermine	102.1162	6.9						0.23			0.30																					
C08306	Hexylamine	102.1279	12.1																														
C01672	Cadaverine	103.1229	7.8	3.8	28.7	2.1	5.9	0.57	38.8	3.9	0.56	9.0	3.6	1.5	9.5	0.61	2.5	0.6	3.9	1.0	0.49	1.7	3.6	2.4	16.8	3.0	5.8	0.60	12.6	2.2	0.54	11.4	3.2
C00334	GABA	104.0705	12.0	0.63	0.9	0.39	0.83	0.23	3.0	0.68	0.36	1.5	0.49	0.29	0.43	0.19	0.38	0.16	0.46	0.20	0.23	0.46	0.38	0.53	0.67	0.70	1.1	0.23	1.2	0.40	0.31	2.4	0.49
C05145	3-Aminoisobutyrate	104.0710	12.3	0.61	0.32	0.19	0.31	0.35	0.81	0.60		0.52		0.41	0.16		0.10	0.31	0.17	0.16		0.21		0.50	0.24	0.22	0.34	0.34	0.35	0.36		0.61	
C02356	2AB	104.0708	15.1	0.22	1.7	0.32	1.1	0.16	11.9	0.57	0.35	0.55	0.22	0.19	0.45	0.07	0.45	0.19	0.69	0.19	0.31	0.32	0.31	0.24	0.81	0.41	1.2	0.18	1.6	0.30	0.33	0.88	0.29
C02721	N-Methylalanine	104.0705	16.1																														
C01026	N,N-Dimethylglycine	104.0710	17.1						0.44																								
C00114	Choline	104.1069	10.7	3.3	4.2	4.5	6.2	2.4	7.8	4.3	2.6	9.2	3.5	1.9	2.5	1.5	3.5	2.4	2.4	1.8	2.3	2.6	4.0	3.9	7.2	7.5	9.9	4.5	4.8	3.3	4.5	13.2	5.2
C06393	2,3-Diaminopropionate	105.0658	11.2																														
C00065	Ser	106.0498	15.7	2.7	6.0	2.7	4.0	1.0	19.7	4.0	2.0	8.0	3.8	1.5	2.3	0.7	1.8	0.9	2.0	1.1	1.3	1.9	2.4	8.3	19.0	10.2	14.9	2.3	18.4	8.0	5.0	19.2	11.5
C06772	Diethanolamine	106.0862	12.0	4.5	5.9	3.6	4.5	3.7	3.1	2.3	3.6	4.0	3.7	7.9	4.3	4.1	4.2	9.7	2.6	4.2	3.4	4.5	10.6	3.1	3.9	14.9	3.9	6.0	7.1	7.3	3.4	2.6	3.4
C02299	N-Methylaniline	108.0806	11.6																														
C00519	Hypotaurine	110.0268	28.5	1.2					1.5	0.80	0.87			0.8					0.66			0.58							0.77		0.9		
C01987	2-Aminophenol	110.0601	12.2																														
C00380	Cytosine	112.0504	11.3																														
C00388	Histamine	112.0863	7.4	0.16	0.23	0.20	0.10		2.5	1.9		0.19	0.19	0.19	0.10	0.25			0.66	0.9			0.41	0.17		0.23			1.2	0.84	0.10	0.13	0.16
C00106	Uracil	113.0359	34.3			4.2	5.1		6.6	4.9															6.9	8.8	8.5		3.9	4.4		5.7	
C00386	Carnosine	114.0602	10.6	0.10																				0.10			0.11					0.23	
C00791	Creatinine	114.0661	11.4	3.8	3.7	2.1	3.8	2.3	2.8	2.7	2.4	3.1	2.8	1.3	1.1	0.61	1.3	1.0	0.67	0.8	1.4	0.56	1.6	3.9	3.7	2.3	3.9	2.4	2.6	3.0	2.7	2.9	3.0
C08311	Muscimol	115.0504	12.1																														
C02512	beta-Cyanoalanine	115.0502	22.2																														
C00148	Pro	116.0707	17.0	19.2	131	7.6	17.9	4.3	60.6	56.7	12.6	24.4	17.7	8.1	40.0	1.0	8.2	3.0	4.3	7.3	8.1	5.3	15.3	23.4	112	49.0	44.9	5.9	68.4	83.4	17.2	105	47.1
C00581	Guanidinoacetate	118.0608	12.9																														
C00431	5-Aminovalerate	118.0865	12.6	177	399	75.4	250	49.3	953	409	102	238	145	68	124	12.0	72	22.8	66.6	77.1	67	40.8	188	84.1	181	56.4	174	23.0	319	175	55.0	316	164
C00183	Val	118.0861	15.7	1.4	29.0	4.0	11.0	0.78	122	12.8	2.0	3.8	2.1	0.7	5.2	0.41	2.5	0.42	5.4	1.2	1.7	1.2	2.1	2.0	10.1	6.7	9.2	1.2	18.8	5.5	2.0	10.0	3.5
C00719	Betaine	118.0860	17.7	2.2	2.3	2.1	2.1	1.5	4.7	2.3	1.8	3.6	1.6	1.7	1.0	0.7	1.1	1.0	0.78	0.63	1.3	0.78	1.4	3.2	3.7	3.3	5.3	2.0	3.2	1.1	2.5	11.6	2.3
C02009	Benzimidazole	119.0599	11.2																														
C03283	2,4-Diaminobutyrate	119.0815	10.8																														
C03678	4-Amino-3-hydroxybutyrate	120.0653	12.7																														
C00263	Homoserine	120.0662	15.8	0.17	0.42				0.80	0.33		0.45			0.23										0.28		0.29		0.26			0.60	0.23
C02115	Alpha-Methylserine	120.0656	16.2						0.31																								
C00188	Thr	120.0655	16.5	0.73	1.4	1.1	1.3	0.32	32.0	2.2	0.63	2.4	0.78	0.49	0.6	0.26	0.7		1.03	0.40	0.53	0.76	0.6	1.2	3.3	2.7	4.5	0.55	6.1	1.5	1.0	5.1	1.6
C15587	Purine	121.0515	12.7																														
C01262	Anserine	121.0684	10.7								0.34										0.25										0.35		
C00884	Homocarnosine	121.0682	10.8																														
C01784	Benzamidine	121.0761	12.0																														
C00097	Cys	122.0272	17.7																														
C09815	Benzamide	122.0571	33.4	2.6	14.1	2.9	9.1	4.0	42.2	14.3	6.5	15.8	6.5	2.0	5.2		3.2	2.4	4.2	3.7	3.1	4.7	7.9		7.2	3.5	4.8	4.9	8.9	4.5	4.3	14.3	9.1
C05332	Phenethylamine	122.0953	12.1						0.18																								
C02455	alpha-Methylbenzylamine	122.0962	12.5																														
C11003	2,4-Dimethylaniline	122.0963	12.7																														
C02421	Isonicotinamide	123.0552	11.5																														
C00153	Nicotinamide	123.0551	11.6	0.16	0.30	0.46	0.25	0.14	0.38			0.49			0.17		0.17			0.15	0.25	0.20	0.26	0.26				0.15			0.37		0.26
C01950	Picolinamide	123.0550	16.2																														
C02567	N1-Acetylspermine	123.1203	8.7																														
C02635	3-Chloroalanine	124.0150	20.3																			0.076											
C00245	Taurine	126.0226	34.4	34.7	50.4	23.1	48.6	14.7	47.3	40.0	21.6	111	26.1	18.0	26.7	14.4	27.6	14.5	23.0	13.4	16.6	30.5	21.4	28.6	43.2	35.6	66.6	20.8	38.8	25.0	25.9	135	37.2
C02376	5-Methylcytosine	126.0661	12.2																														
C05127	1-Methylhistamine	126.1011	7.7						0.09	0.038									0.027							0.045			0.071				
C02835	Imidazole-4-acetate	127.0501	12.5																														
C00178	Thymine	127.0462	34.4			2.1	4.1	1.1	15.5			8.6													8.4	3.1	5.1		4.9	4.4		5.1	4.3
C08737	Melamine	127.0723	11.9																														
C03969	1-Amino-1-cyclopentanecarboxylate	130.0860	15.0																														
C00408	Pipecolate	130.0862	16.1	0.47	0.45	0.17	0.14		0.83	0.24	0.073	0.42	0.13		0.14								0.10	0.33	0.31	0.27			0.35	0.18	0.058	0.51	
C07151	Metformin	130.1088	8.8																														
C01740	Octylamine	130.1594	13.1																														
C02714	N-Acetylputrescine	131.1178	13.2	0.73	1.9	0.37	1.0	0.29	4.1	1.6	0.40	1.1	0.51	0.45	0.8	0.09	0.37	0.21	0.41	0.35	0.28	0.18	0.5	0.60	2.4	1.1	2.2	0.30	2.1	0.9	0.50	2.3	1.2
C00179	Agmatine	131.1284	8.0	0.10	0.13	0.068	0.10		0.25	0.15	0.060	0.13	0.061	0.047	0.08		0.051		0.027	0.036	0.07	0.042	0.11	0.16	0.54	0.32	0.19	0.047	0.18	0.11	0.15	0.23	0.18
C01015	Hydroxyproline	132.0668	18.9	0.44	0.51	0.34	0.44	0.18	0.35	0.63	0.28	0.77			0.17		0.24	0.20		0.26	0.40	0.31		0.37		0.41	0.59	0.27	0.22	0.43	0.55	1.3	
C00430	5-Aminolevulinate	132.0661	12.5						0.17	0.077																			0.09				
C00300	Creatine	132.0766	13.9	4.8	7.7	4.3	6.6	2.4	2.8	6.9	3.8	9.1	6.3	2.6	3.2	1.7	3.6	2.0	2.5	2.1	2.9	2.6	3.8	6.2	8.3	6.8	11.8	3.9	5.2	5.4	5.7	14.5	6.0
C02378	6-Aminohexanoate	132.1020	13.1																														
C02486	beta-Leucine	132.1020	13.7																														
C00407	Ile	132.1021	16.0	0.39	9.5	1.2	3.5	0.18	37.3	4.7	0.40	1.2	0.50	0.33	1.2		0.51	0.08	1.2	0.26	0.28	0.23	0.5	0.66	4.1	2.5	3.4	0.30	7.7	3.2	0.37	3.7	1.1
C00123	Leu	132.1022	16.2	1.4	23.3	3.2	10.4	0.85	73.8	11.6	2.3	4.9	2.9	0.6	4.2	0.16	2.2	0.28	2.5	0.9	1.2	0.77	1.8	2.0	10.1	6.3	9.1	1.0	16.5	6.0	1.9	10.0	3.6
C03375	Bis(3-aminopropyl)amine	132.1497	6.8																														
C02037	Gly-Gly	133.0605	13.1	0.52	1.1	0.42	1.0	0.22	2.7	0.9	0.22	0.37	0.28	0.33	0.27		0.43	0.09	0.16		0.08			0.43	1.3	1.8	1.4	0.18	2.2	0.66	0.17	1.2	0.69
C00152	Asn	133.0589	16.4					0.59					0.64																0.33				
C00077	Ornithine	133.0970	10.7	4.2	20.0	7.9	8.8	1.9	21.2	34.7	5.0	13.3	5.5	6.1	10.8	2.2	7.4	3.5	3.2	2.8	4.2	1.9	7.7	11.4	42.5	19.7	33.1	7.6	37.6	40.0	12.9	40.3	26.4
C00049	Asp	134.0449	18.1	5.1	11.8	12.6	6.4	2.1	19.6	15.2	4.3	12.0	4.9	1.9	2.3	1.3	2.6	1.7	1.8	2.1	2.9	1.5	3.9	16.1	45.6	27.1	34.2	9.8	13.5	18.9	16.6	30.5	22.1
C10901	2-Aminobenzimidazole	134.0713	11.7																														
C00155	Homocysteine	136.0427	16.5																														
C00147	Adenine	136.0620	11.9	0.33	0.45	0.62	0.35	0.080	1.2	0.46	0.13	0.76	0.20	0.13	0.19	0.19	0.11	0.057	0.17	0.076	0.17	0.20	0.23	0.81	0.86	1.0	0.83	0.47	0.79	0.84	0.76	0.79	0.52
C00262	Hypoxanthine	137.0448	17.4	0.70	1.1	1.6	1.3		9.0	1.3		3.5	0.9	0.6		0.51	0.24		0.60			0.85	1.8	1.8	4.9	5.3	5.0	0.84	2.9	1.2	1.0	5.2	3.9
C02918	1-Methylnicotinamide	137.0710	11.5																														
C00568	p-Aminobenzoate	138.0547	15.2																														
C01004	Trigonelline	138.0529	16.4									0.25										0.09								0.072		0.22	
C00108	Anthranilate	138.0526	16.9	0.23	1.1		0.24		0.67	0.63	0.18	0.28	0.24	0.11	0.44		0.12			0.12			0.09	0.23	1.0	0.40	0.39	0.11	0.72	0.9	0.25	0.9	0.50
C02735	Phenylethanolamine	138.0908	12.8																														
C00483	Tyramine	138.0912	13.0				0.28					1.1					0.12					0.50					0.64					0.57	
C00785	Urocanate	139.0494	13.0			1.1			1.5			1.1												1.1	4.1	6.1	4.4		4.0	1.2	0.9	2.4	3.2
C01020	6-Hydroxynicotinate	140.0335	35.3																														
C00783	Tropinone	140.1067	11.8																														
C00860	Histidinol	142.0966	8.3						0.082																								
C00729	Tropine	142.1224	12.6																														
C06231	Ectoine	143.0815	14.8																														
C04294	4-Methyl-5-thiazoleethanol	144.0474	12.6																														
C10172	Proline betaine	144.1027	18.1	0.073			0.21	0.40									0.08	0.21									0.40	0.30				0.18	
-	N1,N12-Diacetylspermine	144.1250	11.8						0.37	0.073		0.083												0.09	0.19		0.23		0.16	0.10	0.074	0.38	0.11
C01035	gamma-Guanidinobutyrate	146.0922	13.0																														
C01996	Acetylcholine	146.1177	11.9																														
C01181	gamma-Butyrobetaine	146.1177	12.6	0.63	1.5	0.60	1.7	0.28	7.6	2.1	0.75	1.5	0.51	0.37	0.52	0.16	0.50	0.19	0.52	0.38	0.42	0.24	0.5	0.38	1.2	0.63	1.9	0.35	2.5	1.1	0.62	2.9	0.81
C01930	Lysinamide	146.1286	8.8																														
C00315	Spermidine	146.1649	7.0	0.37	0.78	0.66	0.56	0.029	8.4	1.6	0.17	1.0	0.20	0.08	0.31	0.06	0.28	0.051	0.21	0.15	0.14	0.20	0.43	0.63	2.1	1.0	1.5	0.25	1.2	0.57	0.66	2.6	0.87
C00064	Gln	147.0765	16.9	4.3	15.0	3.5	5.6	1.6	8.2	19.9	1.9	9.5	3.0	1.5	9.0	1.1	3.1	1.0	3.3	2.5	2.3	2.7	3.9	7.4	34.0	12.4	19.1	3.5	21.1	18.1	5.5	34.1	12.3
C03114	5,6-Dimethylbenzimidazole	147.0915	12.5																														
C00047	Lys	147.1131	10.8	9.2	38.7	7.2	9.7	3.9	63.7	21.7	5.3	24.2	6.8	5.6	11.8	1.7	6.2	4.3	3.2	3.2	3.0	2.5	5.7	14.2	57.3	25.0	34.1	8.8	50.0	39.1	11.5	55.8	25.8
C07166	Trientine	147.1600	6.5																														
C00025	Glu	148.0607	17.2	8.6	48.9	13.9	21.4	3.2	62.0	26.3	3.7	17.5	5.2	4.4	23.8	3.1	9.3	2.1	6.9	4.4	3.9	4.5	4.2	21.8	63.2	46.2	53.0	13.9	55.7	19.5	14.3	68.8	19.5
C00979	O-Acetylserine	148.0600	19.8						0.48																								
C06524	Nornicotine	149.1066	8.1																														
C00073	Met	150.0584	16.8						30.5	1.1				0.53			0.55									1.1	1.0		2.9		0.52	0.89	1.6
C00913	3-Methyladenine	150.0774	12.1																														
C08434	6-Methylaminopurine	150.0773	12.7																														
C00242	Guanine	152.0568	13.0	0.40	0.60	0.61	0.39	0.21	1.0	0.39	0.22	1.2	0.47	0.22	0.13	0.33	0.15	0.11	0.24		0.20	0.42	0.32	0.78	1.8	1.1	0.81	0.70	0.86	0.80	0.84	0.58	1.1
C06818	Amantadine	152.1409	13.5																														
C00385	Xanthine	153.0387	30.5																														
C02518	4-Aminosalicylate	154.0496	20.4																														
C00632	3-Hydroxyanthranilate	154.0498	17.0																														
C03758	Dopamine	154.0859	13.6																														
C05135	4-(beta-Acetylaminoethyl)imidazole	154.0967	13.2						0.25	0.083	0.034								0.054	0.040									0.09	0.045			
C10865	Pseudopelletierine	154.1227	12.4																														
C00135	His	156.0769	11.4	3.9	7.7	3.8	4.8	1.8	4.4	8.5	2.6	8.3	4.2	3.7	5.3	2.1	6.2	2.9	1.9	2.3	2.7	1.9	4.1	3.6	12.8	8.8	19.5	3.1	10.4	9.3	5.5	15.9	10.9
C05568	beta-Imidazolelactate	157.0618	13.8						0.30																				0.13				
C10600	Ibotenate	159.0401	28.6																														
C01551	Allantoin	159.0552	34.3	16.0	17.9	18.2	19.5	11.8	14.7	15.6	16.2	16.7	17.9	16.2	14.2	16.2	18.1	13.4	14.5	15.3	13.3	15.9	14.7	12.8	13.6	14.5	18.4	15.6	15.8	17.2	16.7	11.2	17.7
C00637	Indole-3-acetaldehyde	160.0727	34.6						22.4																								
C08269	Betonicine	160.0968	20.6																														
-	N-Acetylvaline	160.0966	35.3																														
C00993	Ala-Ala	161.0911	14.5	0.40	0.52	0.42	0.49	0.11	4.2	0.46	0.079	0.52	0.20	0.29	0.30	0.12	0.34	0.17	0.33	0.11	0.14	0.18	0.26	0.49	1.0	0.9	1.7	0.17	1.7	0.36	0.35	1.5	0.54
C00398	Tryptamine	161.1070	13.0																														
C00956	alpha-Aminoadipate	162.0759	17.2	0.49	0.51	0.25	0.71	0.17	2.2	0.62	0.28	0.80	0.37	0.25	0.25		0.36	0.19	0.36	0.19	0.26	0.26	0.34	0.75	0.79	0.36	1.1	0.37	0.72	0.66	0.48	1.3	0.61
C01046	N-Methylglutamate	162.0758	19.8																														
C00955	Indole-3-ethanol	162.0910	34.5																														
C00318	Carnitine	162.1124	13.3	0.34	0.56	0.50	0.60	0.23	0.48	0.45	0.31	0.82	0.39	0.22	0.26	0.19	0.36	0.18	0.30	0.20	0.27	0.24	0.40	0.62	1.2	1.1	1.7	0.63	0.9	0.86	1.1	1.6	0.87
C02627	2-Deoxystreptamine	163.1076	9.4																														
C16741	5-Hydroxylysine	163.1074	11.2																														
C00745	Nicotine	163.1226	8.3																														
C00715	Pterin	164.0565	16.0																														
C03172	S-Methylmethionine	164.0732	11.4																														
C02989	Methionine sulfoxide	166.0518	18.6						0.89																				0.26				
C02242	7-Methylguanine	166.0719	12.9	0.085		0.072	0.089		0.21	0.082		0.087			0.049				0.055					0.081	0.16	0.10			0.12			0.15	
C02230	3-Methylguanine	166.0722	13.1																														
C00079	Phe	166.0864	17.4	6.8	37.5	7.3	14.2	4.1	48.5	15.7	4.3	21.0	5.4	4.6	11.9	2.0	7.2	3.0	4.9	2.6	2.6	2.6	3.9	5.3	20.9	11.2	19.9	3.4	22.0	9.2	3.8	19.4	7.3
C01959	Taurocyamine	168.0404	34.2		1.6	4.1		4.1				3.0					2.8	2.8			2.2		4.7	3.6	2.7	4.6	3.1					3.4	
C00250	Pyridoxal	168.0661	13.7						0.20																								
C05587	3-Methoxytyramine	168.1017	13.8																														
C04548	Synephrine	168.1020	14.0																														
C00534	Pyridoxamine	169.0973	9.3																														
C00314	Pyridoxine	170.0813	13.5																														
C00547	Noradrenaline	170.0811	14.2																														
C01152	3-Methylhistidine	170.0922	11.7	0.11	0.11		0.13	0.11	0.27	0.12	0.16	0.19					0.06	0.08	0.068	0.046	0.12	0.047		0.083	0.080	0.12	0.19	0.11	0.12		0.21	0.27	
C11016	Diphenylamine	170.0964	31.2																														
C02693	Indole-3-acetamide	175.0861	34.4																														
C00437	N-Acetylornithine	175.1078	15.0						1.9																				0.42				
C01047	N-gamma-Ethylglutamine	175.1081	17.9		0.25		0.25		0.88	0.18		0.38			0.20		0.12		0.13						0.12		0.28		0.31			0.50	
C00062	Arg	175.1192	11.1	12.6	18.7	13.7	9.8	3.9	0.83	7.1	5.4	24.8	12.3	2.4	1.6	1.2	2.8	6.1	1.2	1.9	3.1	2.8	7.9	35.0	54.8	24.3	38.5	19.8	5.8	23.9	21.3	31.8	42.7
C08304	Gramine	175.1229	13.2																														
C06213	N-omega-Methyltryptamine	175.1227	13.4																														
C03139	Guanidinosuccinate	176.0664	15.8																														
C00954	Indole-3-acetate	176.0715	34.7		1.4		1.4		3.3	2.0	0.9	1.2								0.54	0.7	0.52					1.0		0.89	0.65		1.0	0.73
C10898	2-Guanidinobenzimidazole	176.0926	12.8																														
C00327	Citrulline	176.1032	17.3	2.1	8.1	4.8	2.2	1.0	4.8	8.0	1.3	6.3	2.4	1.9	3.0	0.8	1.6	1.1	0.67	0.9	1.2	0.68	2.8	6.5	36.7	26.3	23.9	4.0	14.3	11.3	4.1	30.7	16.7
C00308	Canavanine	177.0981	11.6																														
C00780	Serotonin	177.1022	13.7																														
C08265	Alliin	178.0530	22.1																														
C01419	Cys-Gly	179.0484	14.4																														
-	Xanthopterin	180.0510	20.7																														
C03570	Mannosamine	180.0862	14.3																														
C00329	Glucosamine	180.0861	14.5						5.1																0.47		0.35		0.51			1.0	
-	n-Butyl a-Picolinate	180.1017	16.4																														
C03510	Methionine sulfoximine	181.0639	13.2																														
C00082	Tyr	182.0813	17.8	6.0	22.0	6.9	15.0	3.4	34.7	18.4	6.5	31.2	10.8	4.6	8.4	2.1	9.2	3.7	2.2	2.7	4.3	3.6	6.3	8.0	29.1	16.8	33.9	6.3	21.3	17.7	10.9	34.9	20.6
C09209	Harman	183.0916	13.5																														
C00588	Phosphorylcholine	184.0735	32.1	2.7	5.4	2.0	5.0	2.9		5.2	5.0	4.0	6.0	1.5	2.2	1.0	2.0	2.8	1.17	2.1	2.4	1.2	2.5	3.0	3.6	1.7	4.5	2.7	2.2	5.2	4.2		4.0
C00788	Epinephrine	184.0965	14.6																														
C04259	N-alpha,N-alpha-Dimethylhistidine	184.1074	13.0																														
C01029	N8-Acetylspermidine	188.1745	10.0						0.42																				0.12			0.17	
C00612	N1-Acetylspermidine	188.1749	9.9	0.19		0.14	0.084		2.0	0.14	0.089	0.45	0.051	0.11		0.061	0.052		0.35			0.17	0.12	0.32		0.61	0.30	0.10	1.0		0.22	0.9	0.053
C02155	Gly-Leu	189.1237	15.3	0.11	0.27	0.29	0.28		3.7	0.58		0.33		0.16	0.08		0.17							0.19	0.59	0.59	0.76		1.1	0.27	0.11	1.2	0.46
C02727	N-epsilon-Acetyllysine	189.1235	17.9						0.46																								
C03793	N6,N6,N6-Trimethyllysine	189.1576	11.2						0.16																				0.068				
C02256	Castanospermine	190.1070	14.3																														
C05659	5-Methoxytryptamine	191.1177	13.8																														
C05635	5-Hydroxyindoleacetate	192.0623	34.8																														

C00021	SAH	193.0684	13.7																														
C03752	Glucosaminate	196.0806	18.7																														
C00355	DOPA	198.0763	18.3																														
C02997	N-Acetylhistidine	198.0867	15.4						0.10																							0.10	
C00019	SAM+	200.0764	11.1				0.053		0.56				0.11											0.078	0.13	0.15	0.18		0.11			0.12	0.10
C01404	Arginine ethyl ester	203.1501	9.6																														
C03626	ADMA	203.1477	12.0						0.13																								
-	SDMA	203.1500	12.2																														
C02571	o-Acetylcarnitine	204.1231	14.2	0.31	0.68	0.28	0.58	0.21		0.20	0.44	0.62	0.74	0.12	0.19	0.11	0.20	0.22	0.16	0.14	0.24	0.15	0.31	0.39	0.86	0.63	0.82	0.45	0.15	0.36	0.85	0.61	0.61
C00078	Trp	205.0971	17.3	0.22	1.6	0.47	0.69		5.9	1.3	0.15	0.84	0.22	0.12	0.49	0.12	0.32		0.22	0.12	0.14	0.11	0.25	0.26	1.0	0.87	1.4		1.2	0.39	0.19	1.1	0.81
C05660	5-Methoxyindoleacetate	206.0756	34.5	1.1		1.1	1.0	0.75	1.3	1.0	1.1	1.3	1.0	1.1	1.0	0.9	1.1	1.0	1.01	0.9	0.7	0.9	1.1	1.0		0.87	0.89	1.1	1.0	1.1	1.1	1.0	1.1
C00328	Kynurenine	209.0924	15.5																														
-	Cysteine-glutathione disulphide	214.0505	18.3																														
C01909	Desthiobiotin	215.1380	34.6																														
C05341	beta-Ala-Lys	218.1494	10.5																														
C01118	o-Succinylhomoserine	220.0808	35.6																														
C00371	trans-Zeatin	220.1189	15.1																														
C00643	5-Hydroxytryptophan	221.0920	17.8																														
C01239	N-Acetylglucosylamine	221.1115	15.6																													0.38	
C00140	N-Acetylglucosamine	222.0983	34.4	7.5	18.8	8.1	8.5	2.8	181	9.1	2.7	15.4	13.7	1.8	1.4	0.8	2.5	3.9	4.1	1.5	2.3	2.3	11.2	15.8	16.7	7.3	15.8	15.2	17.6	6.9	8.7	9.2	38.1
C02291	Cystathionine	223.0752	15.5																														
C02794	3-Hydroxykynurenine	225.0867	15.3																														
C00881	2'-Deoxycytidine	228.0979	14.8						0.086	0.072															0.19				0.081	0.13			
-	N1,N8-Diacetylspermidine	230.1857	16.1		0.041	0.044	0.035		0.84	0.10	0.025	0.10			0.017				0.035	0.032			0.026	0.068	0.14	0.19	0.18	0.049	0.25	0.087	0.053	0.31	0.15
-	gamma-Glu-2AB	233.1135	19.7																														
C01598	Melatonin	233.1276	34.3																														
C00835	Sepiapterin	238.0935	28.4																														
C02953	7,8-Dihydrobiopterin	240.1082	17.2																														
C00491	Cystine	241.0303	17.3	0.11	0.17				0.57			0.15													0.09	0.17	0.22					0.21	
C00884	Homocarnosine	241.1293	10.8																														
C03592	5-Methyl-2'-deoxycytidine	242.1131	15.3																														
C00214	Thymidine	243.0959	34.5				2.3		2.8	2.3							1.8				2.0								2.6	1.9			
C00475	Cytidine	244.0937	15.2	0.26	0.38	0.41	0.31	0.090	0.74	0.18	0.12	0.56	0.19	0.11	0.11	0.10	0.10	0.10	0.14	0.09	0.16	0.30	0.33	0.37	0.27	0.14	0.16	0.28	0.31	0.34	0.49	0.17	0.20
C00299	Uridine	245.0852	34.4	2.3	2.3	2.6	3.3		19.9	2.9	1.5	5.1	2.2			2.0	1.8		2.5	1.7	1.7	1.3	1.9	2.9	2.9	3.7	3.1	2.8	4.4	2.3	2.7	3.5	3.9
C04137	Octopine	247.1305	16.9			0.44	0.22		1.8	0.61		0.25												0.30	0.9	0.9	0.71	0.21	1.4	0.45	0.32	0.77	0.82
C00647	Pyridoxamine 5'-phosphate	249.0631	16.4																														
C00669	gamma-Glu-cys	251.0700	20.1																														
C06470	Muramate	252.1077	17.2																														
C05198	5'-Deoxyadenosine	252.1097	15.1						0.14	0.050															0.14				0.037	0.073			
C01736	Purine riboside	253.0931	19.8																														
C05512	2'-Deoxyinosine	253.0913	28.4																														
C04874	7,8-Dihydroneopterin	256.1029	17.5																														
C00670	Glycerophosphorylcholine	258.1075	34.1										0.80																				0.51
C00378	Thiamine	265.1111	10.3																														
C03795	N6-Methyl-2'-deoxyadenosine	266.1244	15.7																														
C00212	Adenosine	268.1041	15.5	0.078	0.054	0.13	0.11	0.13	0.58	0.042	0.11	0.056		0.044	0.048	0.054	0.048	0.053	0.061		0.09	0.12		0.20	0.19	0.16	0.068	0.16	0.12	0.13	0.20	0.054	0.067
C00330	2'-Deoxyguanosine	268.1038	18.0						0.35	0.11															0.59	0.29			0.21	0.16			
C01817	Homocystine	269.0586	15.1																														
C00294	Inosine	269.0865	30.5			0.35			1.8			0.54												0.42	0.68	0.77		0.38			0.27		0.29
C01425	Glu-Glu	277.1031	17.1	0.28	0.67	0.32	0.37		1.6	0.72		0.25	0.18	0.22	0.20		0.19							0.32	0.77	0.73	0.66		1.3	0.52	0.16	0.56	0.55
C00449	Saccharopine	277.1336	16.9	0.17	0.17				0.45																0.26	0.19			0.27				0.20
C02494	1-Methyladenosine	282.1191	15.6																														
C00387	Guanosine	284.0997	19.6	0.14		0.16	0.12	0.12	0.58	0.036		0.26	0.031	0.052								0.15		0.33	0.57	0.39		0.27	0.17	0.24	0.24		0.11
C01762	Xanthosine	285.0826	32.8																														
-	Ophthalmate	290.1349	20.7																														
C03406	Argininosuccinate	291.1298	14.8																														
C00170	5-Methylthioadenosine	298.0966	15.8																														
C00127	Glutathione(ox)	307.0847	19.1									0.083		0.048			0.08	0.07				0.10							0.057				
C01670	N-alpha-Benzenolarginine ethylester	307.1761	16.5																														
C02515	3-Iodotyrosine	307.9775	18.9																														
C00051	Glutathione(red)	308.0913	20.8																														
-	Phe-Phe	313.1553	17.1	0.033		0.038	0.036		0.10	0.079		0.16											0.06	0.043	0.18	0.086	0.14		0.071	0.09	0.039	0.09	0.10
C04677	AICAR	339.0818	34.7	0.65		0.58	0.9		1.6	0.59	0.74		0.64	0.45	0.6		0.7	0.51	0.45									0.78	0.62	0.72	0.67		0.77
C01081	Thiamine monophosphate	345.0782	16.8																														
C02890	Tetrahydropalmatine	356.1860	17.7																														
C03958	TRH	363.1777	16.9																														
C00255	Riboflavin_mzgap	377.0948	34.4	4.9	3.1	2.4	11.0	4.0	0.65	1.8	7.4	1.0	5.8	1.5	1.7	1.3	3.8	2.3	1.6	1.1	1.7	0.49	1.1	2.8	2.4	2.4	5.2	5.6	3.3	2.2	5.1	0.65	2.9
C03451	S-Lactoylglutathione	380.1121	21.6																														
C11331	Leu-Leu-Tyr	408.2500	18.7																														
C01060	3,5-Diiodo-tyrosine	433.8755	19.7																														
C00440	5-Methyltetrahydrofolate	460.2069	18.8	2.2	2.4	4.2	2.7		7.6	6.5		3.1	2.1										2.1	3.3	4.8	5.9	7.7		6.2	3.7		4.0	4.8
C02465	3,3',5-Triiodothyronine	651.7985	21.9																														
C00048	Glyoxylate	72.9950	11.7																														
C00163	Propionate	73.0304	12.2	145	549	86.1	345	47.6	1716	509	118	420	122	61	172	14.5	101	27.8	109.8	91	90	103	172	54.2	264	37.6	212	27.7	311	144	64.1	474	142
C00160	Glycolate	75.0093	11.2	3.6	3.7	3.8	6.8	5.4	7.5	3.5	5.9	4.8	4.4	1.4	3.2	2.6	2.7	2.0	2.0	1.5	3.6	2.3	2.6	3.5	2.8	3.5	4.2	6.0	3.6	4.7	5.9	4.6	3.5
C01771	Crotonate	85.0297	12.7		3.7	1.6	3.5		31.9	9.1	1.6	3.9	4.0		0.8						1.8		9.0									9.8	
C00022	Pyruvate	87.0094	11.1	12.9	32.5	19.8	30.0	6.7	280	2.9	14.5	40.4	27.2	11.1	13.4	5.4	18.8	9.6	37.3	3.1	15.1	10.5	34.7	41.7	73.6	33.7	105	32.9	122	5.9	43.3	76.6	77.6
C00246	Butyrate	87.0456	13.1	16.2	49.3	14.0	71.9	13.6	233	51.8	31.4	99	39.2	5.3	11.5	3.1	10.7	5.4	12.5	10.1	21.1	19.0	42.5	6.7	16.7	8.5	18.8	6.8	27.0	14.2	12.1	89.8	12.9
C00186	Lactate	89.0252	12.3	38.3	89.4	49.6	110	48.0	348	24.4	46.1	94	69.1	27.4	30.9	39.2	52.3	46.7	141	22.4	63	78.6	53	45.8	91	64.9	105	84.6	122	37.3	74.4	95	84.6
C01013	3-Hydroxypropionate	89.0248	12.5	9.2	9.5	6.0	7.8	2.6	9.4	11.3	5.0	7.6	8.9	6.8	7.5	4.2	6.5	3.3	9.7	3.8	4.0	3.8	3.5	9.9	20.4	6.7	13.8	4.8	13.4	9.1	6.3	7.0	14.9
C11145	Methanesulfonate	94.9817	10.2																														
C08279	Tiglate	99.0461	13.2																														
C00109	2-Oxobutyrate	101.0249	12.1	0.66	0.85		1.4		5.5	3.0	0.48	0.62	0.73		0.6		0.6				0.28		1.4	1.7	8.2		1.0	1.6	0.53	0.9	0.77	1.6	1.1
C00803	Pentanoate + 3-Methylbutanoate	101.0610	13.8	8.9	22.7	8.2	18.8	2.4	45.8	17.2	5.3	25.1	6.2	3.6	6.9	1.6	5.8	1.2	4.3	4.0	3.9	3.6	9.0	4.0	12.8	5.7	11.0	1.3	11.5	5.6	3.1	26.7	8.4
C00383	Malonate	103.0041	8.7	1.3	2.0	1.1	1.2	1.1	6.2	1.7	1.1	1.5	1.4	1.7	1.4	0.9	1.1	0.8	1.6	1.1	1.0	1.1	1.3	1.1	1.5	1.3	1.3	1.0	2.5	1.3	1.3	1.6	1.6
-	2-Hydroxyisobutyrate	103.0407	13.0																														
C05984	2-Hydroxybutyrate	103.0400	13.2	0.37	0.89	0.72	1.7	0.79	4.7		0.57		0.66		0.31		0.7	0.48	0.71		0.42	0.30	0.5	2.1	4.3	1.4	2.0	3.4	3.3	1.3	1.1	0.81	0.85
C01089	3-Hydroxybutyrate	103.0400	13.4	2.1	6.0	2.4	4.2	1.0	8.5	4.6	1.6	2.5	1.7	1.2	4.2	1.4	2.1		1.8	1.4	1.0	1.3	1.7	3.3	27.1	4.8	5.5	1.7	4.7	5.2	1.8	3.4	2.0
C03173	(Methylthio)acetate	105.0003	12.4																														
C00258	Glycerate	105.0201	12.6																														
C05942	Pyrrole-2-carboxylate	110.0254	12.7																														
C02704	Methyl sulfate	110.9764	10.1																														
C01546	2-Furoate	111.0092	12.6																														
C00122	Fumarate	115.0040	9.1	4.3	1.8	1.5	1.3	3.2	4.2	2.0	1.1	1.8	2.3	4.3	4.1	1.4	4.3	1.6	0.95	4.5	1.9	1.6	4.8	1.9	3.5	2.0	2.1	4.5	4.6	1.2	1.1	1.4	4.4
C00141	2-Oxoisopentanoate	115.0396	12.7		0.45	0.41	0.65	0.29	3.5	0.64		0.44	0.84		0.45	0.18	0.40	0.28	0.35		0.28		0.6	1.0	1.4	0.42	0.60	0.72	1.1	0.41	0.9	0.58	0.68
-	4-Oxopentanoate	115.0409	13.2																														
C01585	Hexanoate	115.0767	14.2	8.9	2.6	8.6	25.2	2.2	67.5	24.9	6.6	8.8	5.7	3.9	1.4	1.5	6.0	0.9	5.1	4.6	4.6	1.8	10.9	3.4	1.8	4.5	10.6	0.89	13.3	5.3	3.5	11.0	8.2
C00042	Succinate	117.0197	9.4	20.6	79.7	23.1	57.6	6.3	242	57.3	8.0	14.2	19.6	6.4	33.3	6.7	28.2	4.8	18.0	12.4	6.0	12.2	12.5	14.7	72.3	24.9	41.7	6.4	90	45.5	7.5	20.3	11.9
-	2-Hydroxypentanoate	117.0556	13.8	0.58	1.5	0.60	2.6	0.65	14.0	1.9	0.74	1.1	1.4	0.42	0.6	0.40	1.4	0.29	1.6	0.20	1.2	0.61	1.3	0.56	1.7	1.0	2.3	0.86	3.1	1.1	1.0	1.3	1.1
C00180	Benzoate	121.0303	13.1																40.9														
C00253	Nicotinate	122.0264	13.1			0.80			4.2	0.89		0.89					0.37		3.5						1.9	2.3	2.6		1.3		0.58	3.0	1.2
C03782	1-Aminoethylphosphonate	124.0178	15.6																														
C03557	2-Aminoethylphosphonate	124.0177	16.3																														
C05123	Isethionate	124.9906	11.7		0.25		0.22		0.37	0.16		0.37													0.35		0.28		0.13			0.27	
C00813	Barbiturate	127.0156	13.2																														
C01879	5-Oxoproline	128.0356	13.5	2.8	8.6	4.0	3.8	1.6	7.6	4.6	1.7	3.4	3.3	2.0	4.2	1.7	3.3	2.2	1.9	1.7	2.1	3.1	2.7	4.4	7.0	6.1	6.3	2.1	6.8	3.7	2.5	4.9	5.3
C00490	Itaconate	129.0206	9.7																														
C02226	Citraconate	129.0204	10.0																														
C00233	4-Methyl-2-oxopentanoate	129.0548	13.4	0.47	1.6	1.1	1.1	0.36	7.6	1.3	0.69	0.67	0.9	0.36	0.9	0.24	0.9	0.41	0.62	0.58	0.51	0.26	0.9	1.2	2.7	1.4	1.6	0.9	3.5	1.3	1.1	1.3	1.4
-	4-Oxohexanoate	129.0567	13.8																														
C02129	4-Acetylbutyrate	129.0568	14.0																														
C17714	Heptanoate	129.0923	14.7			0.17		0.11								0.20			0.15	0.16		0.18	0.19			0.19							
C01073	N-Acetyl-beta-alanine	130.0505	14.2	0.37				0.29	1.4	0.49		0.64	0.39									0.27					0.59		0.79			0.79	
C00489	Glutarate	131.0359	10.0																														
C02642	3-Ureidopropionate	131.0470	13.7																														
C03264	2-Hydroxy-4-methylpentanoate	131.0717	14.4		1.7	0.69	3.4		28.7	2.6					0.7		1.2		1.09				0.7		2.4	1.0	2.8		3.7	1.7		1.0	0.74
C06103	6-Hydroxyhexanoate	131.0720	14.8																														
C00711	Malate	133.0143	9.4	1.5	2.2	2.5	2.2	1.4	3.6	2.1	1.5	2.3	2.4	1.1	1.2	1.2	1.5	1.5	1.12	1.0	1.5	1.7	1.6	2.8	4.6	3.0	2.2	3.0	2.9	1.7	2.5	2.4	2.7
C01620	Threonate_mzgap	135.0376	13.8	2.1	22.3	2.4	8.9	2.1	29.1	17.4	3.8	21.3	3.6	1.1	6.1	0.7	2.5	0.9	2.3	3.1	3.0	3.1	4.7	2.0	10.6	2.9	6.3	2.0	7.9	5.0	2.7	21.6	5.0
C00805	o-Hydroxybenzoate	137.0251	12.6																														
C00156	p-Hydroxybenzoate	137.0249	13.6																														
C00587	m-Hydroxybenzoate	137.0253	13.8																														
C05682	Phosphonoacetate	138.9809	9.6																														
C00169	Carbamoylphosphate	139.9759	9.9																														
C00346	Ethanolamine phosphate	140.0124	15.9	13.8	9.0	4.7	33.2	30.3			26.7	9.8	67.6	4.4	1.8	8.2	5.3	18.7	3.0	3.1	10.5	5.3	13.5	4.0		0.90		16.8		1.6	10.5		3.1
C02595	2-Thiopheneacetate	141.0030	13.5																														
C06423	Octanoate	143.1072	15.2																									0.32			
C00026	2-Oxoglutarate	145.0121	9.3	1.0	3.4	2.9	1.4	0.88	4.9	0.87	0.87	2.1	2.9	1.1	4.0	1.4	2.9	0.9	1.10	0.62	1.2	0.37	2.5	6.6	8.5	5.4	4.3	6.2	6.4	2.0	3.7	4.1	4.3
C06104	Adipate	145.0500	10.5	0.36	0.59	0.30	0.64	0.29	0.80	0.68	0.23	0.39	0.54	0.29	0.6	0.48	0.54	0.7	0.48	0.53	0.7	0.9	0.5	0.33	0.44	0.34	0.47	0.22	0.55	0.26	0.23	0.45	0.44
C03618	threo-beta-methylaspartate	146.0464	14.4																														
C01180	4-Methylthio-2-oxobutyrate	147.0126	13.1																														
C02630	2-Hydroxyglutarate + Citramalate	147.0303	10.1	1.4	4.3	2.5	3.2	0.54	5.2	4.3	1.0	2.0	1.2	1.4	3.2	1.0	2.3	1.0	1.8	1.4	1.2	1.1	1.5	2.6	8.5	6.1	7.7	1.7	5.5	5.3	2.0	5.9	3.4
C00423	trans-Cinnamate	147.0510	14.5	0.54	3.0	0.66	1.0		3.8	1.4		1.5	0.37	0.45	1.1	0.47	0.47		0.41	0.21	0.26	0.28	0.38	1.7	3.4	1.8	2.5	0.78	2.7	0.88	1.0	2.4	1.0
C00898	Tartrate	149.0102	9.3																														
C02137	Benzoylformate	149.0250	13.1																														
C05629	3-Phenylpropionate	149.0613	14.5	12.1	4.8	12.3	26.5	1.9	65.3	18.2	7.1	9.7	7.3	4.4	1.7	1.6	7.0	0.7	5.0	4.2	4.2	1.5	11.3	5.2	3.1	6.3	13.5	1.3	14.5	5.8	3.6	9.2	9.4
C05852	2-Hydroxyphenylacetate	151.0411	14.1																														
C00642	p-Hydroxyphenylacetate	151.0404	14.6		15.6		7.4	2.3	14.9	13.8		25.8	3.8		3.9		2.8			2.8	1.8	3.3	2.8		6.2		5.2		2.1	6.8		26.6	3.4
C00606	Cysteine sulfinate	152.0032	13.5		1.1	0.85			2.0		0.72	1.2		0.53	0.9		0.54		0.76							1.1	1.4		1.3			2.0	
C00628	2,5-Dihydroxybenzoate	153.0199	13.5		2.6						1.1				0.6										1.3						0.72		
C00295	Orotate	155.0091	13.1	0.51					2.0	0.73	0.24		0.52		0.37										1.2		1.0		0.9			1.8	0.57
C03030	2,4-Dihydroxypyrimidine-5-carboxylate	155.0115	13.4																														
C00337	Dihydroorotate	157.0259	13.6																														
-	2-Oxooctanoate	157.0878	14.4																														
C01601	Pelargonate	157.1245	15.7													0.7	0.33	0.27	0.35	0.30	0.30	0.37											
C00322	2-Oxoadipate	159.0310	9.9																														
C02656	Pimelate	159.0672	10.9																														
-	2-Hydroxyoctanoate	159.1034	15.4																														
C01044	N-Formylaspartate	160.0265	9.9																														
C03761	3-Hydroxy-3-methylglutarate	161.0450	10.4	0.17					0.34	0.20		0.20							0.081										0.26			0.20	
C00166	Phenylpyruvate	163.0398	13.9						1.7																	0.73			1.4				0.75
C00811	p-Coumarate	163.0411	14.5																														
C01772	o-Coumarate	163.0410	14.6																														
C06337	Terephthalate	165.0199	10.1																														
C01606	Phthalate	165.0198	10.5	0.42	0.49	0.39	0.40	0.40	0.33	0.31	0.27	0.43	0.59	0.41	0.42	0.35	0.30	0.42	0.36	0.30	0.52	0.50	0.34	0.44	0.44	0.32	0.39	0.41	0.41	0.49	0.27	0.40	0.45
C05607	3-Phenyllactate	165.0547	14.6	0.64	2.3	1.0	2.6	0.33	6.1	2.0	0.47	0.9	0.70	0.6	1.2	0.53	1.2	0.44	0.68	0.60	0.41	0.33	0.6		3.9	1.5	2.7		2.6	1.2	0.52	1.3	1.1
C01198	3-(2-Hydroxyphenyl)propionate	165.0563	14.8																														
C01744	3-(4-Hydroxyphenyl)propionate	165.0546	15.2	3.6	5.8	2.9	19.4		12.8	12.1	3.6	20.2	3.9		3.5		6.1		2.9	3.1	2.4	2.6	4.6		4.2		10.6		3.7	4.4		15.8	3.9
C03722	2,3-Pyridinedicarboxylate	166.0153	10.3																														
C00074	PEP	166.9764	9.4	0.30				0.21	0.80	0.30		0.41		0.19	0.17				0.19		0.16			0.52	0.75	0.68	1.0	0.40	0.78	0.47	0.33	1.4	0.57
C00366	Urate	167.0218	14.2	138	171	117	360	208	22.9	56.2	209	50.5	174	51.0	54.0	38.4	106	100	38.6	25.9	71	8.6	51	137	131	112	313	170	116	60.5	156	27.4	92
C11527	4-Hydroxymandelate	167.0358	14.6	0.67	10.7	1.4	5.6	1.3	9.9	10.1	1.4	16.7	1.8	0.34	2.8		2.2	0.54	0.75	2.0	1.0	2.4	2.0		5.1	1.3	4.2	0.69	0.39	3.6	1.3	16.8	2.2
C00506	Cysteate	167.9984	12.2	0.27	0.76		0.37		0.68						0.31		0.25		0.27						0.56		0.65		0.63	0.26		0.32	0.38
C05672	2-Amino-3-phosphonopropionate	168.0077	11.2																														
C00111	DHAP	168.9917	11.2	2.9	5.0	4.1	3.5	1.3	4.0	2.4	2.4	9.6	4.8	1.5	1.1	1.0	1.0	1.1	1.8	0.73	1.8	1.9	4.7	4.6	6.1	5.7	8.9	3.4	4.3	2.3	6.5	7.8	10.4
C00661	G3P	168.9919	11.7																														
C00093	Glycerophosphate	171.0071	11.5	3.9	5.9	4.9	7.0	2.3	5.2	2.8	5.1	5.8	14.8	1.3	1.1	0.9	1.8	1.7	1.5	1.5	2.5	1.4	4.4	5.0	5.0	4.5	7.5	3.9	3.6	3.1	4.6	3.2	8.3
C01571	Decanoate	171.1401	16.2																														
C06325	2-Quinolinecarboxylate	172.0414	14.5																														
C02710	N-Acetylleucine	172.0990	15.9		0.14				0.27	0.17																							
C02734	Phenyl phosphate	173.0021	11.2																														
C02341	trans-Aconitate	173.0105	8.7																														
C00417	cis-Aconitate	173.0097	8.9	0.22	0.45	0.23	0.25	0.25	0.055	0.16	0.23	0.53	0.82	0.12	0.24	0.11	0.16	0.26	0.097		0.39	0.24	0.20	0.53	0.82	0.20	0.22	0.63	0.15	0.45	0.71	0.11	0.79
C00493	Shikimate	173.0556	15.5			0.66			3.2	1.0				0.37	0.50								0.6		1.9	2.1	2.0		2.2	1.0		2.5	1.2
C01042	N-Acetylaspartate	174.0417	10.6	0.79	1.9	0.53	1.2	0.58	2.0	0.67	0.41	0.87	1.9	0.32	0.43	0.17	0.27	0.28	0.32	0.27	0.18	0.18	0.5	2.5	3.7	0.9	1.7	1.1	2.2	2.1	0.9	1.2	1.8
C00438	Carbamoylaspartate	175.0359	10.4						1.2																								
C00499	Allantoate	175.0482	14.5																								1.0				
C02504	2-Isopropylmalate	175.0610	10.8	0.59	0.33	0.27	0.20	0.12	0.43	0.20	0.13	0.30	0.18	0.27	0.22	0.19	0.16	0.14	0.22	0.21		0.13	0.18	0.43	0.36	0.53	0.34	0.16	0.79	0.26	0.20	0.59	0.32
C03145	N-Formylmethionine	176.0394	14.7																														
C01586	Hippurate	178.0525	15.0																														
C05582	Homovanillate	181.0505	15.4	1.6	5.6	1.8	6.3	1.0	4.0	6.8	1.6	3.0	2.4	1.1	3.0	1.1	2.4	0.9	0.97	2.0	1.2	0.9	2.1	0.68	3.5	1.5	7.1	1.0	2.6	4.3	1.9	3.5	3.9
C00847	4-Pyridoxate	182.0455	14.5																											0.049	
C02703	Serine O-sulfate	183.9926	12.2																														
C01005	O-Phosphoserine	184.0021	11.4	0.17	0.20	0.24	0.17		1.4	0.39		0.23												0.21	1.0	0.60	0.79		0.59	0.29		1.4	0.28
C00631	2PG + 3PG	184.9866	9.6	0.36	0.33	0.52	0.35		1.4	0.61	0.20	0.65		0.32	0.19		0.25	0.22	0.32		0.21		0.22	1.0	1.7	1.5	1.5	0.59	1.2	0.85	0.64	1.6	1.0
C17715	Undecanoate	185.1557	16.8																														
C08261	Azelate	187.0980	11.5	0.11	0.15	0.16		0.15	0.067	0.16		0.10	0.17	0.15	0.25	0.17		0.15		0.11	0.17	0.15		0.26		0.20	0.24	0.060		0.077	0.18		0.13
C02774	10-Hydroxydecanoate	187.1341	16.7											0.20	0.16	0.16	0.13	0.15	0.18	0.14	0.12	0.15											
C00624	N-Acetylglutamate	188.0573	11.0	0.089	0.14	0.089	0.12	0.069	0.74	0.19		0.20	0.11		0.13				0.12	0.09			0.08	0.11	0.29	0.23	0.38	0.10	0.33	0.19	0.09	0.69	0.17
C05829	N-Carbamylglutamate	189.0529	10.8																														
C02712	N-Acetylmethionine	190.0557	15.6						0.72																								
C00311	Isocitrate	191.0195	8.9	0.30	0.77			0.55			0.48	0.73	0.9		0.41								0.37	0.54	1.5	0.31	0.46	1.0	0.37	0.64	1.0		1.2
C00158	Citrate	191.0206	9.0	5.9	12.7	6.3	7.8	15.6	0.24	0.15	9.6	11.4	23.1	0.8	0.34	1.0	0.47	3.2	0.52	0.36	3.0	1.6	4.2	21.6	24.3	2.0	1.5	29.4	0.24	5.1	24.2	1.0	18.4
C00296	Quinate	191.0574	15.4																														
C05598	Phenaceturate	192.0675	15.4																														
C00191	Glucuronate	193.0352	15.7																				0.76					0.45		0.60	
C01494	trans-4-Hydroxy-3-methoxycinnamate	193.0510	15.2																														
C07588	o-Hydroxyhippurate	194.0463	12.8																														
C00257	Gluconate	195.0522	15.6																														
C10833	Syringate	197.0466	15.0																														
C05584	4-Hydroxy-3-methoxymandelate	197.0468	15.5																														
C02679	Dodecanoate	199.1715	17.9																														
C05824	Cysteine S-sulfate	199.9705	12.2																														

C08277	Sebacate	201.1139	11.8					0.12						0.044							0.040	0.041											
C11284	3-Indolebutyrate	202.0885	15.9																														
C02470	Xanthurenate	204.0312	11.4																														
C03519	N-Acetylphenylalanine	206.0832	16.0		0.19				0.43	0.22					0.13																		
C09816	Benzylsuccinate	207.0676	11.3																														
C00818	Saccharate	209.0313	10.6																														
C00879	Mucate	209.0310	10.7	0.24		0.21	0.18	0.24		0.10	0.39	0.30	0.24										0.17	0.60	0.35	0.45	0.23	0.84	0.23	0.30	0.73	0.44	0.46
-	3-Indoxyl sulfate	212.0039	13.5																														
C00672	2-Deoxyribose 1-phosphate	213.0179	12.2						3.1	0.66															1.1	0.63	0.53		1.0			1.0	0.42
C00864	Pantothenate	218.1034	16.7		0.18				0.53																				0.20			0.30	0.10
C00482	Sinapate	223.0626	16.1																														
C00931	Porphobilinogen	225.0894	17.1																														
C00199	Ru5P	229.0129	12.1	1.9	2.0	3.1	1.2	0.61	12.2	2.0	0.61	3.3	1.4	0.49	0.50		0.52		0.60		0.39	0.49	0.9	5.3	8.1	6.8	3.4	2.8	5.2	2.8	3.2	2.0	4.0
C00117	R5P	229.0116	12.4	0.61	0.79	0.80	0.55	0.24	2.1	0.83		1.5	0.67	0.32	0.30		0.47	0.14		0.22	0.54	0.42	0.7	1.1	2.1	2.2	1.7	0.59	1.6	0.70	1.0	1.1	1.8
C02678	Dodecanedioate	229.1445	12.4											0.051	0.040	0.041		0.046		0.041	0.035	0.036											
C06369	2-Deoxyglucose 6-phosphate	243.0291	12.8																														
C00120	Biotin	243.0827	16.8					0.30		0.45	0.61		0.72								0.39				0.58								
C00352	Glucosamine 6-phosphate	258.0394	14.3																														
C00103	G1P	259.0232	12.9	0.16	0.11	0.29	0.17	0.14	3.4	0.11		0.20		0.32	0.13	0.09							0.17	0.087	0.33	1.4	1.2	0.47	1.0	0.29	0.82	0.67	1.4
C00085	F6P	259.0236	13.0		0.70	0.30	0.16		0.73	0.48		0.76			0.22	0.23	0.33		0.18				0.5	0.72	1.2	0.79	0.49	0.37	0.62		0.58	0.62	0.73
C00092	G6P	259.0231	13.1	0.75	1.3	1.9	1.3	0.20	0.13	3.1	0.47	4.0	0.48	0.53	0.6	0.15	1.0	0.7	0.81	0.55	1.1	0.60	1.9	1.7	2.0	2.8	5.6	0.52	1.7	1.1	2.7	2.9	5.1
C01096	Sorbitol 6-phosphate	261.0394	12.9																														
C01159	2,3-DPG	264.9536	9.8																														
C04037	Galacturonate 1-phosphate	273.0032	10.8																														
C00345	6-Phosphogluconate	275.0170	10.6		0.30	0.19			0.34			0.78	0.21										0.28	0.19	0.44	0.40	0.83	0.15	0.27	0.21	0.32	1.1	2.2
C05382	S7P	289.0331	13.3		0.76	0.53				0.80		0.9							0.49		0.43		0.5		1.7	2.0	2.0		2.1		0.80	1.4	1.8
C02713	N-Acetylmuramate	292.1023	18.0														0.26																
C04501	N-Acetylglucosamine 1-phosphate	300.0490	13.4	0.18	0.21	0.22	0.22		0.78	0.30		0.47	0.20	0.10				0.10					0.21		0.38	0.37	0.51		0.53	0.37	0.33	0.58	0.58
C00357	N-Acetylglucosamine 6-phosphate	300.0501	13.8			0.18			2.9																								
C02354	2',3'-cCMP	304.0359	16.8																														
C00239	dCMP	306.0511	13.2																														
C00365	dUMP	307.0350	13.0																														
C00270	N-Acetylneuraminate	308.1002	18.2	32.4	79.0	43.4	28.4	7.4	42.6	26.9	27.0	65.8	58.8	11.0	12.2	2.5	8.0	9.8	39.5	11.1	18.6	5.8	59	34.4	43.4	24.8	39.4	30.9	32.0	24.1	34.9	46.2	128
C01182	Ribulose 1,5-diphosphate	308.9799	10.3																														
C00968	3',5'-Cyclic dAMP	312.0524	16.8																														
C00364	dTMP	321.0520	13.4							0.10																							
C00055	CMP	322.0457	13.3																			0.16									0.15		0.21
C00105	UMP	323.0306	13.1																														
C00575	cAMP	328.0465	17.1																														
C01204	Phytate	328.9260	10.3																														
C00943	cIMP	329.0304	15.9																														
C00360	dAMP	330.0617	13.6																														
C00354	F1,6P	338.9872	10.6						0.27															0.24	0.36		0.39	0.23	0.13	0.16	0.30	0.38	0.35
C00942	cGMP	344.0418	16.9																														
C01367	3'-AMP	346.0569	13.2																														
C00020	AMP	346.0578	13.7																														
C00130	IMP	347.0416	13.4																														
C00584	Prostaglandin E2	351.2169	19.5																														
C00639	Prostaglandin F2alpha	353.2346	19.6																														
C00144	GMP	362.0523	13.9																														
C00655	XMP	363.0364	11.3																														
C01103	Orotidine 5'-monophosphate	367.0199	11.3																														
C02273	Digalacturonate	369.0689	13.4																														
C00005	NADPH	371.5403	11.9																														
C00010	CoA	382.5524	12.4																														
C00705	dCDP	386.0174	11.7																														
C01346	dUDP	387.0015	11.6																														
C00119	PRPP	388.9462	10.1																														
C00363	dTDP	401.0175	11.9																														
C00112	CDP	402.0126	11.8																														
C00015	UDP	402.9962	11.7																														
C00024	Acetyl CoA	403.5580	12.7																														
C00695	Cholate	407.2814	19.9																														
C00206	dADP	410.0280	12.1																														
C01260	P1, P4-Di(adenosine-5') tetraphosphate	417.0192	12.4																														
C00630	Isobutyryl CoA	417.5728	12.9																														
C00689	Trehalose 6-phosphate	421.0773	15.0																														
C00083	Malonyl CoA	425.5533	11.6																														
C00224	Adenosine 5'-phosphosulfate	426.0133	12.8																														
C00054	Adenosine 3',5'-diphosphate	426.0233	11.0																														
C00008	ADP	426.0241	12.2																														
C00104	IDP	427.0078	12.1																														
C00091	Succinyl CoA	432.5603	11.7																														
C00504	Folate	440.1343	12.8																														
C00035	GDP	442.0190	12.4																														
C03794	Adenylosuccinate	462.0680	10.8																														
C01921	Glycocholate	464.3025	20.5																														
C00458	dCTP	465.9846	11.4																														
C00460	dUTP	466.9678	11.3																														
C00459	dTTP	480.9842	11.5																														
C00063	CTP	481.9792	11.4																														
C00075	UTP	482.9632	11.3																														
C00131	dATP	489.9949	11.8																														
C01345	dITP	490.9779	11.7																														
C00002	ATP	505.9902	11.7																														
C00286	dGTP	505.9892	11.9																														
C00081	ITP	506.9726	11.6																														
C05122	Taurocholate	514.2858	20.5																														
C00044	GTP	521.9857	11.9																														
C00700	XTP	522.9690	10.6																														
C00307	CDP-choline	547.1230	19.9																														
C00301	ADP-ribose	558.0643	14.8																														
C00842	TDP-glucose	563.0685	14.8																														
C00029	UDP-glucose	565.0490	14.6																														
C00167	UDP-glucuronate	579.0279	12.1																														
C00498	ADP-glucose	588.0768	15.1																														
C00096	GDP-mannose	604.0715	15.3																														
C00043	UDP-N-acetylglucosamine	606.0745	14.9																														
C00128	CMP-N-acetylneuraminate	613.1422	15.5																														
C00003	NAD+	662.1036	21.1																														
C00857	Deamido-NAD+	663.0879	15.2																														
C00004	NADH	664.1190	15.4																														
C00006	NADP+	742.0697	13.5																														
C00016	FAD	784.1522	16.1																														

Red font : conc>500uM

S/N <10

Orange font : Neutral compounds
